# Higher hemoglobin levels are associated with adverse heart rate variability in a middle‐aged birth cohort

**DOI:** 10.14814/phy2.70406

**Published:** 2025-06-06

**Authors:** Samuli Sakko, Mikko. P. Tulppo, Peppi Koivunen, Joona Tapio

**Affiliations:** ^1^ Research Unit of Extracellular Matrix and Hypoxia, Biocenter Oulu and Faculty of Biochemistry and Molecular Medicine University of Oulu Oulu Finland; ^2^ Research Unit of Biomedicine and Internal Medicine University of Oulu Oulu Finland; ^3^ Medical Research Center Oulu University Hospital and University of Oulu Oulu Finland

**Keywords:** baroreflex, cardiovascular disease, heart rate variability, hemoglobin, smoking

## Abstract

High hemoglobin (Hb) levels within normal variation range are considered beneficial. However, lower Hb levels within normal variation range are associated with healthier cardiovascular traits. Heart rate variability (HRV) and baroreflex sensitivity (BRS) are dysregulated in cardiovascular diseases (CVDs). We have shown in a hypertensive cohort that higher Hb levels are associated with impaired HRV and BRS. As CVDs are exacerbated by aging, conditions like hypertension and factors such as smoking, further studies on the association of Hb levels and HRV and BRS on younger and healthier populations are required for the generalization of these associations. The aims were to cross‐sectionally study the association of Hb levels with HRV and BRS in the Northern Finland Birth Cohort 1966 (NFBC1966) at 46 years (*n* = 5342) and to evaluate confounding factors, such as smoking, on these associations. Higher Hb levels within normal variation range were associated with adverse time‐domain measures, including elevated heart rate (HR). Hb levels were negatively associated with high‐frequency (HF) power and positively with the low frequency (LF) to HF ratio (LF/HF). These associations were influenced by sex, metabolic parameters, and smoking but were observed regardless of these factors. For BRS, adjusting for metabolic covariates nullified the association with Hb levels.

## 1 INTRODUCTION

Heart rate variability (HRV) is regulated by the autonomic nervous system (ANS) and its individual components represent aspects of autonomous cardiac function (Shaffer & Ginsberg, 2017). Baroreflex, measured as baroreflex sensitivity (BRS), is an autonomous function responsible for the short‐term regulation of blood pressure (BP) and heart rate (HR) (La Rovere et al., 2008). Decreased HRV and BRS are associated with a spectrum of cardiometabolic diseases such as key components of metabolic syndrome, including type 2 diabetes (Benichou et al., 2018) and hypertension (Huikuri et al., 1996) and with various clinical outcomes, including heart failure and myocardial infarction (Chattipakorn et al., 2007).

Hemoglobin (Hb) is the principal carrier of oxygen in the circulatory system. Hb levels are regulated by genetic (sex, ethnicity) (Van Der Harst et al., 2012) environmental (altitude) (Van Der Harst et al., 2012) and age‐related factors (Stauder & Thein, 2014), and influenced by lifestyle factors (smoking) (Malenica et al., 2017), diseases (e.g., anemia) and certain medications. Fluid balance and dehydration can alter Hb levels significantly (Jimenez et al., 1999). Nevertheless, Hb levels remain stable throughout adult life and are utilized, for example, as an identifier in biological passports (Lobigs et al., 2016).

Higher Hb levels within the normal variation range are associated with key components of metabolic syndrome, including hypertension (Auvinen et al., 2021), obesity (Auvinen et al., 2021), non‐alcoholic fatty liver disease (Yu et al., 2012), and insulin resistance (Facchini et al., 1998) and with cardiovascular outcomes such as stroke (Panwar et al., 2016) and CVD mortality (Tapio et al., 2021). Recently, we showed in a Finnish middle‐aged hypertensive cohort (aged 40–59 years, *n* = 733, 51.7% hypertensives) that higher Hb levels within the normal variation range were associated with decreased HRV and BRS (Tapio et al., 2023). However, due to the impact of factors such as antihypertensive medication on HRV (Schroeder et al., 2003), these results are prone to confounding. Additionally, factors such as aging and smoking status affect both Hb levels (Malenica et al., 2017; Stauder & Thein, 2014) and HRV (Hayano et al., 1990). Therefore, studies on larger and healthier populations, with more control over the confounding effects, are necessary to enhance reliability and generalization of the results (Kahlert et al., 2017).

Thus, our aims here were (1) to cross‐sectionally evaluate the effect of Hb levels within normal variation on HRV and BRS measures utilizing data from the Northern Finland Birth Cohort 1966 (NFBC1966) at the age of 46 (*n* = 5342, 15.1% hypertensives) and (2) to evaluate the effect of confounding factors (sex, smoking, and antihypertensive medication) on these associations.

## 2 METHODS

### 2.1 Study population

**FIGURE 1:**
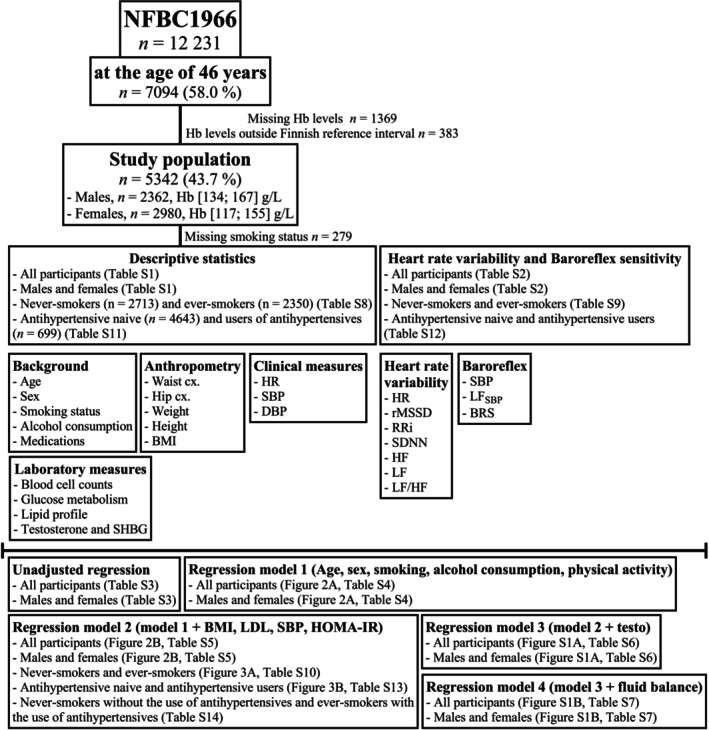
Flow‐chart of the study population. BMI, body mass index; BRS, baroreflex sensitivity; cx, circumference; DBP, diastolic blood pressure; Hb, hemoglobin; HF, high‐frequency; HOMA‐IR, homeostatic model assessment for insulin resistance; HR, heart rate; LDL, low‐density lipoprotein; LF, low‐frequency; LF/HF, low‐frequency‐to‐high frequency ratio; LFSBP, low‐frequency SBP; n, number of; rMSSD, root mean square of successive differences in RRi; RRi, R‐R interval; SBP, systolic blood pressure; SBP, systolic blood pressure; SDNN, standard deviation of normal‐to‐normal interbeat intervals; SHBG, sex‐hormone binding globulin; testo, testosterone; WH, waist‐to‐hip.

Figure 1 presents a flow‐chart of the study. The NFBC1966 is a prospective cohort comprising all children in the two northernmost provinces of Finland, with expected dates of delivery between 1st of January and 31st of December 1966 (12,058 live births). The latest data collection was conducted at 46 years of age (University of Oulu, 2020), when all subjects with a known postal address in Finland were invited to participate in clinical examinations, which included background questionnaires, laboratory measures, and clinical evaluation, including HRV and BRS measures (Nordström et al., 2021).

Of the 7094 participants that attended the 46‐year data collection, those with Hb levels within the Finnish reference interval (*n* = 5342, males [134; 167 g/L] and females [117; 155 g/L]) (Kairisto et al., 2003) were included in the study. Inclusion criteria for subpopulation analyses were data on smoking status (*n* = 5063) and the use of antihypertensive medication (*n *= 5342).

### 2.2 Background information

Smoking status was categorized as “never‐smokers” or “ever‐smokers” based on self‐report. Alcohol consumption was categorized according to standard drinks per week “0–1”, “2–5”, and “6 or more” based on self‐report. Participants reported their medication use.

Physical activity was measured using a wrist‐worn accelerometer (Polar Electro Oy) as previously described (Niemelä et al., 2019). Daily Metabolic Equivalent (MET) averages were calculated for the following activity levels: 1.00–1.99 MET = sedentary, 2.00–3.49 MET = light, 3.50–4.99 MET = moderate, 5.00–7.99 MET = vigorous, and ≥8.00 MET = very vigorous. The amount of moderate‐to‐vigorous physical activity (MVPA) was used.

### 2.3 Clinical examination

Body weight and height were measured using calibrated digital scales and stadiometers, and BMI was calculated. Waist and hip circumferences were measured, and waist‐to‐hip (WH) ratio was calculated.

Heart rate (HR), brachial systolic blood pressure (SBP), and diastolic blood pressure (DBP) were measured three times at rest while seated, using an appropriately sized cuff and a digital device, and the mean was used in the analysis.

### 2.4 Laboratory measures

The blood samples were taken after an overnight fast, and then immediately centrifuged and analyzed without storing. The blood samples were analyzed in NordLab Oulu testing laboratory (T113) accredited by the Finnish Accreditation Service (FINAS) (EN ISO 15189). Fasting serum insulin (fs‐Insulin, ref. 02230141) and fasting blood glucose (fB‐glucose, ref. 05001429) levels were assessed by radioimmunoassay (Pharmacia Diagnostics, Uppsala, Sweden) and further analyzed using an enzymatic dehydrogenase method (Advia 1800, Siemens Healthcare Diagnostics, Tarrytown, NY, USA) and by a chemiluminometric immunoassay (Advia Centaur XP, Siemens Healthcare Diagnostics, Tarrytown, NY, USA). Homeostatic model assessment of insulin resistance (HOMA‐IR) was used to evaluate insulin resistance. HOMA‐IR was calculated as (fB‐glucose × fs‐insulin/22.5). Total cholesterol (ref 10376501), high‐density lipoprotein (HDL, ref. 07511947) and low‐density lipoprotein cholesterol (LDL, ref. 09796248), and triglycerides (ref 09580156) were determined using enzymatic assay methods.

### 2.5 HRV and BRS measurements

Measurements of HRV and BRS have been previously described in detail (Perkiömäki et al., 2016). A HR monitor (RS800CX, Polar Electro Oy, Kempele, Finland) was used to record R‐R intervals (RRi) with an accuracy of 1 ms. Standard lead‐II ECG (Cardiolife, Nihon Kohden, Tokyo, Japan) and BP by finger plethysmography (Nexfin, BMEYE Medical Systems, Amsterdam, the Netherlands) were recorded during the protocol with a sampling frequency of 1000 Hz (PowerLab 8/35, ADInstruments). Breathing frequency was spontaneous during HRV and BRS measurements. Examination time was 6 min, with the first 3 min seated and the last 3 min standing. Examinations were preceded by at least a 1 min stabilization period. The first 150 s while seated and the last 150 s in a standing position were used in the HRV analyses. The R‐R interval (RRi) data were edited based on visual inspection (Hearts 1.2 Software, University of Oulu, Oulu, Finland). Artifacts and ectopic beats were removed and replaced by local average. However, sequences with ≥10 consecutive beats of noise or ectopic beats were deleted. The RRi series with ≥80% accepted data were included in analyses (Perkiömäki et al., 2016). Mean HR, root mean square of successive differences in RRi (rMSSD), RRi, standard deviation of normal‐to‐normal interbeat intervals (SDNN), spectral power densities (fast Fourier transform, length 512 beats) at the low‐frequency (LF, 0.04–0.15 Hz, ms^2^) and high‐frequency (HF, 0.15–0.40 Hz, ms^2^) components of HRV and their ratio (LF/HF) were calculated.

For the BRS analysis, a fast Fourier transform (Welch method) was performed to analyze the LF power of RRi and SBP oscillations (LF ms^2^, LF_SBP_ mmHg^2^) for subsequent analysis of BRS by the alpha method if sufficient coherence (≥0.5) between LF oscillations in RRi and SBP was verified (Kiviniemi et al., 2013). Out of 2726 recordings, BRS was successfully calculated for 2641 participants while seated and for 2617 participants while standing.

### 2.6 Statistical methods

Continuous variables were evaluated for skewness and kurtosis and log‐transformed if skewed. Normally distributed continuous variables are presented as mean (M) and standard deviation (SD) and non‐normally distributed continuous variables as median (Mdn) and interquartile range (IQR). Count data is presented as the number of observations (*n*) and percentage (%). First, analyses were conducted for all participants, followed by subgroup analyses (restriction) based on sex, smoking status, and the use of any antihypertensive medication (including beta‐blockers). Subjects with missing data were omitted from individual analyses. For comparison between two groups, one‐way ANOVA was employed for normally distributed variables and the Mann–Whitney *U* test for non‐normally distributed variables.

Multiple linear regression models were used to assess associations between Hb levels and HRV and BRS. After unadjusted models, the associations were adjusted for sex, smoking, alcohol consumption, age, and MVPA (Model 1). To account for the confounding of metabolic factors, Model 2 was further adjusted for BMI, SBP, LDL cholesterol, and HOMA‐IR. Due to interactions with Hb levels, Model 3 was adjusted additionally for testosterone, and Model 4 further with albumin levels. The 95% confidence intervals (CIs) for effect sizes were estimated by running regression analyses with Z‐scores as standardized predictor variables. All the models were checked for potential multicollinearity of the predictor variables. Data was analyzed using IBM SPSS Statistics software (version 29).

## 3 RESULTS

### 3.1 Higher Hb levels within the normal variation range are associated with adverse time‐ and frequency‐domain measures of HRV

Background characteristics of the study population are presented in Table S1 and HRV and BRS measures in Table S2. First, unadjusted associations between Hb levels and measures of HRV and BRS were evaluated in both seated and standing positions (Table S3). The results were consistent regardless of posture or sex, with Hb levels being positively associated with HR and LF/HF ratio and negatively with rMSSD, RRi, SDNN, HF power, and BRS (Table S3).

**FIGURE 2:**
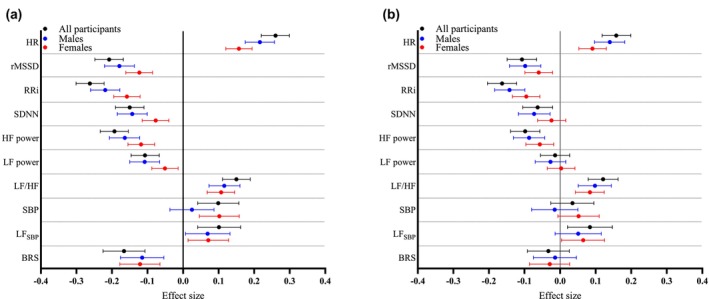
Associations of Hb levels within normal variation range with measures of heart rate variability and baroreflex sensitivity with and without adjusted for metabolism in all participants, males and females. Forest plot representing the effect size estimates and their 95% CIs for 1 SD change in the parameter of interest per 1 SD change in Hb in all participants (black), males (blue), and females (red). The effect sizes were adjusted for (a) sex, age, smoking status, alcohol consumption, and physical activity, and (b) additionally for BMI, LDL cholesterol, SBP and HOMA‐IR. BMI, body mass index; HOMA‐IR, homeostatic model assessment for insulin resistance; LDL, low‐density lipoprotein.

Next, we assessed the association between Hb levels and HRV and BRS measures using sex‐, age‐, smoking‐, MVPA, and alcohol consumption‐adjusted linear regression models (Model 1, Table S4, Figure 2a). Hb levels were positively associated with HR and negatively associated with rMSSD, RRi, and SDNN (Figure 2a). Out of the frequency‐domain measures, Hb levels were negatively associated with HF and LF power and positively associated with the LF/HF ratio (Figure 2a). For SBP, a positive association with Hb levels was observed in the analysis for all participants and females, while for LF_SBP_, a positive association with Hb levels was detected regardless of sex (Figure 2a). For BRS, a negative association with Hb levels was detected regardless of sex (Figure 2a).

As both Hb levels (Auvinen et al., 2021) and HRV (Stuckey et al., 2014) are associated with metabolic conditions, the regression model was further adjusted with key metabolic parameters: BMI, LDL cholesterol, SBP, and HOMA‐IR (Model 2, Figure 2b, Table S5). The effect sizes were slightly attenuated, yet Hb levels were positively associated with HR and negatively with rMSSD and RRi. Of the frequency domain measures, Hb levels were negatively associated with HF power and positively with LF/HF ratio. For LF_SBP_, a positive association with Hb levels was detected in all participants and females. After adjusting for key metabolic parameters, no associations between Hb levels and BRS were detected (Figure 2b).

As Hb levels are positively associated with testosterone levels (Bachman et al., 2014) and regulated by fluid balance (Jimenez et al., 1999), we further adjusted Model 2 with testosterone (Model 3, Figure S1A, Table S6) and albumin levels (Model 4, Figure S1B, Table S7), either of which had little to no effect on the associations.

### 3.2 Smoking and antihypertensive medication impact the association between Hb levels and HRV

**FIGURE 3:**
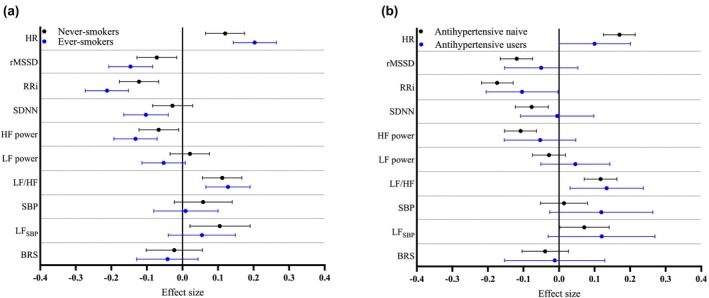
Association of Hb levels within normal variation range with measures of heart rate variability and baroreflex sensitivity according to smoking status and antihypertensive medication use. Forest plot represents the effect size estimates and their 95% CIs for 1 SD change in the parameter of interest per 1 SD change in Hb in (a) never‐smokers (black) and ever‐smokers (blue), and (b) antihypertensive naïve (black) and antihypertensives users (blue). The effect sizes were adjusted for sex, age, smoking status (in case of antihypertensive naïve and antihypertensive users), alcohol consumption, physical activity, BMI, LDL cholesterol, SBP, and HOMA‐IR.

Smoking elevates Hb levels (Malenica et al., 2017) and decreases HRV (Hayano et al., 1990). To control the effect of smoking on the associations, the analyses were conducted separately in never‐smokers and ever‐smokers (Model 2, Figure 3a, Tables S8–S10). Hb levels were positively associated with HR and negatively with rMSSD and RRi regardless of smoking status. Out of frequency‐domain measures, Hb levels were negatively associated with HF power and positively with LF/HF ratio regardless of smoking status. A positive association between Hb levels and LF_SBP_ was only detected in ever‐smokers (Figure 3a).

Antihypertensive drugs have a major effect on HRV (Schroeder et al., 2003), thus analyses were conducted separately based on antihypertensive treatment (Model 2, Figure 3b, Tables S11–S13). In antihypertensive naïve subjects, Hb levels were positively associated with HR and negatively with rMSSD, RRi, and SDNN. Hb levels were negatively associated with HF power and positively with LF/HF ratio in the antihypertensive naïve subjects. The use of antihypertensive medication had a major impact on the associations, as most were undetectable in antihypertensive drug users (Figure 3b).

### 3.3 Association between Hb levels and HRV in a never‐smoking and antihypertensive medication naive population

**FIGURE 4:**
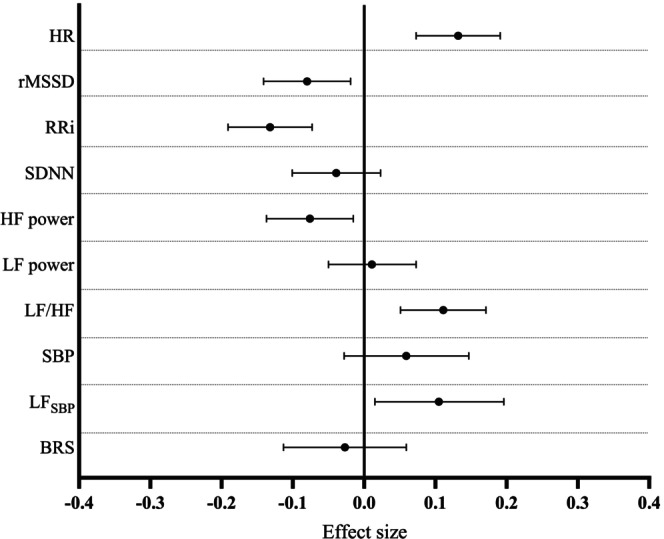
Association of Hb levels within normal variation range with measures of heart rate variability and baroreflex sensitivity in never‐smoking and antihypertensive medication naïve population. Forest plot representing the effect size estimates and their 95% CIs for 1 SD change in the parameter of interest per 1 SD change in Hb. The effect sizes were adjusted for sex, age, smoking status, alcohol consumption, physical activity, BMI, LDL cholesterol, SBP, and HOMA‐IR.

Finally, we studied the association between Hb levels and HRV measures in a never‐smoking and antihypertensive medication naïve population (Model 2, Figure 4, Table S14). In this subpopulation, all the associations observed in all participants in Model 2, except for SDNN, were present. Hb levels were positively associated with HR and negatively associated with rMSSD and RRi. Of the frequency domain measures, Hb levels were negatively associated with HF power and positively associated with LF/HF ratio and LF_SBP_ (Figure 4).

## 4 DISCUSSION

These results demonstrate associations between higher Hb levels and adverse HRV in a Finnish middle‐aged birth cohort. The associations were independent of sex, metabolic parameters, and lifestyle factors, including smoking. The use of antihypertensive medication had a major impact on these associations. These results corroborate our previous findings from a Finnish middle‐aged hypertensive cohort (Tapio et al., 2023), wherein higher Hb levels were associated with decreased HRV. These results provide new insights into the factors influencing the association between Hb levels and cardiovascular autonomic regulation.

Adjusting for metabolic covariates significantly impacted the associations between Hb levels and HRV measures. HF power relates to parasympathetic activity and represents vagal activity (Stuckey et al., 2014). The negative association between Hb levels and HF power was consistent in all models and subpopulation analyses (except for antihypertensive drug users), indicating a reduced vagal activity in subjects with higher Hb levels. The exact meaning of LF power is under debate, as it has been suggested to indicate both parasympathetic and sympathetic activity, as well as baroreflex activity (Goldstein et al., 2011; Martelli et al., 2014; Rahman et al., 2011; Shaffer & Ginsberg, 2017), whereas LF/HF ratio might represent sympathovagal balance, although strongly influenced by measuring conditions. Higher Hb levels were associated with higher LF/HF ratio regardless of covariates, suggesting subjects with higher Hb levels exhibit a dominance of sympathetic activity over vagal activation. Higher LF/HF ratio has been associated with CVDs and CVD‐related outcomes including metabolic syndrome in women (Stuckey et al., 2014), diabetes (Thayer et al., 2010), atherosclerosis, coronary artery disease (Evrengul et al., 2006; Kubota et al., 2017), and CVD events (Fang et al., 2020).

Interestingly, adjusting for metabolic covariates nullified the negative association between Hb levels and BRS, which was observed prior to adjusting with metabolic parameters. The results indicate that the effect of Hb levels on BRS was outweighed by the effect of metabolic parameters on BRS. The results contrast with our prior results, where Hb levels were negatively associated with BRS (Tapio et al., 2023).

The use of antihypertensive medication had a major impact on the associations between Hb levels and HRV. The current study population is a local birth cohort having a controlled setup, whereas our previous cohort is specifically designed to assess CVDs in hypertensive subjects (Tapio et al., 2023). Moreover, the current population is seven‐fold larger, 10 years younger on average, and healthier (13.1% vs. 51.7% hypertensives). A possible mechanism of the association between Hb levels and BRS being observed exclusively in the hypertensive cohort (MacIorowska et al., 2020; Materson et al., 1998; Tacito Yugar et al., 2023) is vascular dynamics. In the baroreflex, baroreceptors detect BP changes signaling the brainstem, which modulates autonomic output, thereby establishing a negative feedback loop to stabilize BP^2^. Healthy individuals exhibit more sensitive vascular regulation, with the peripheral component predominantly in charge, whereas in hypertensive individuals, the ANS component prevails (Mancia & Grassi, 2014). Taken together, Hb levels could be associated with the ANS component of BRS rather than with the peripheral component.

Smoking appeared to amplify the associations between Hb levels and HRV. Ever‐smokers were also metabolically unhealthier compared to never‐smokers, presenting, for example, higher BMI and fasting insulin levels; thus, the observed associations may partially be accounted for by metabolic factors. Nonetheless, higher Hb levels were associated with impaired HRV irrespective of smoking status. This relationship highlights the dual influence of smoking on both Hb levels and HRV, as smoking elevates Hb levels (Malenica et al., 2017) and decreases HRV (Hayano et al., 1990).

The current study population is a well‐defined birth cohort with extensive cross‐sectional data, representing the largest dataset to date examining the association between Hb levels and HRV. The effect of possible confounders was rigorously controlled by (1) excluding extreme Hb levels outside the Finnish reference interval, (2) restriction (by sex, smoking, antihypertensive usage) and (3) using means of multivariate models. Despite these measures, a residual confounding effect may exist. Additionally, the current study employs a short time interval for HRV measures (3 min), limiting comparability to studies with longer time intervals. We present no information on heart failure or atrial fibrillation, which could have had an impact on the data, but considering the relatively young age (46 years) of the study population, these factors are at least partially controlled for by restriction by antihypertensive medication use, a method which also restricts the different impacts antihypertensive drugs have on HRV and BRS depending on the drug class (MacIorowska et al., 2020; Materson et al., 1998; Tacito Yugar et al., 2023). We have not carried out a correction for multiple testing. As the study population is a Finnish birth cohort, these results are most translatable to Caucasian populations.

While no causality or mechanistic data are demonstrated here, the data aligns with the current literature, indicating a complex association between Hb levels, HRV and cardiovascular disease. Likewise, it is important to understand that the associations between Hb levels and HRV are influenced by sex, metabolic factors, smoking and antihypertensive medication. Given the cross‐sectional and observational nature of this study, longitudinal and causal studies are warranted to further elucidate these findings.

## 5 CONCLUSIONS

In this middle‐aged, general population‐based birth cohort, higher Hb levels within the reference interval are associated with impaired HRV, suggesting reduced parasympathetic activity and diminished autonomous cardiac regulation in subjects with higher Hb levels. Collectively, these findings indicate that Hb levels should be considered when assessing cardiovascular health.

## AUTHOR CONTRIBUTIONS

S.S.: Writing the original draft, formal analysis, visualization, review, and editing. M.P.T.: Resources, review and editing, supervision. P.K.: Project administration, supervision, resources, review, and editing. J.T.: Conceptualization, writing the original draft, review and editing, supervision.

## FUNDING INFORMATION

NFBC1966 received financial support from University of Oulu Grant No. 24000692, Oulu University Hospital Grant No. 24301140, and ERDF European Regional Development Fund Grant No. 539/2010 A31592. This study was supported by grants from Research Council of Finland (Project grant 339900 to P.K. and Flagship funding to University of Oulu 357915), Jane and Aatos Erkko Foundation (P.K.), Sigrid Jusélius Foundation (P.K.), and the Finnish Medical Foundation (S.S.).

## CONFLICT OF INTEREST STATEMENT

The authors declare no conflicts of interest.

## ETHICS STATEMENT

The NFBC1966 study adhered to the Declaration of Helsinki and received ethical approval from the Northern Ostrobothnia Hospital District's ethical committee. All participants provided written informed consent. Analyses of the current study were approved by the NFBC project center under project number P1273.

## Supporting information


Appendix S1.

## Data Availability

NFBC data are available from the University of Oulu, Infrastructure for Population Studies. Permission to use the data can be applied for research purposes via an electronic material request portal. In the use of data, we follow the EU general data protection regulation (679/2016) and the Finnish Data Protection Act.
