# Supplementary material for: Higher hemoglobin levels are associated with adverse heart rate variability in a middle‐aged birth cohort
Source: Physiol Rep. 2025 Jun 6;13(11):e70406. doi: 10.14814/phy2.70406 (PMC12141927; doi:10.14814/phy2.70406)
Supplement: Supplementary file 1 — Appendix S1. [file PHY2-13-e70406-s001.docx]

**Higher hemoglobin levels are associated with adverse heart rate variability in a middle-aged birth cohort**

Running head: Hemoglobin and autonomous cardiac regulation

Samuli Sakko^1^, Mikko. P. Tulppo^2,3^, Peppi Koivunen^1^, Joona Tapio^1*^

^1^Research Unit of Extracellular Matrix and Hypoxia, Biocenter Oulu and Faculty of Biochemistry and Molecular Medicine, University of Oulu, P.O. Box 5400, FIN-90014 Oulu, Finland.

^2^Research Unit of Biomedicine and Internal Medicine, University of Oulu, Oulu, Finland.

^3^Medical Research Center, Oulu University Hospital and University of Oulu, Oulu, Finland.

*To whom correspondence should be addressed:

Joona Tapio, email: [joona.tapio@oulu.fi](mailto:joona.tapio@oulu.fi)

P.O. Box 5400 (Aapistie 7 C), FI-90014 University of Oulu, Finland

**Supplementary material**

**Table S1. Characteristics of the study population.**

|  | All participants | | Males | | Females | |  |
| --- | --- | --- | --- | --- | --- | --- | --- |
| Variable (units) | n | M (SD) / Mdn (IQR) | n | M (SD) / Mdn (IQR) | n | M (SD) / Mdn (IQR) | *P* |
| Age (years) | 5342 | 46.6 (0.6) | 2362 | 46.7 (0.6) | 2980 | 46.6 (0.6) | 0.044 |
| Smoking status, n (%) | 5063 |  | 2207 |  | 2856 |  | <0.001 |
| Never-smokers |  | 2713 (53.6) |  | 1051 (47.6) |  | 1662 (58.2) |  |
| Ever-smoker |  | 2350 (46.4) |  | 1156 (52.4) |  | 1194 (41.8) |  |
| Alcohol consumption, n (%) | 5092 |  | 2219 |  | 2873 |  | <0.001 |
| 0-1 |  | 1557 (29.1) |  | 459 (20.7) |  | 1098 (38.2) |  |
| 2-5 |  | 1791 (33.5) |  | 674 (30.4) |  | 1117 (38.9) |  |
| 6 or more |  | 1744 (32.6) |  | 1086 (48.9) |  | 658 (22.9) |  |
| Use of antihypertensives, n (%) | 5342 | 699 (13.1) | 2362 | 331 (14.0) | 2980 | 368 (12.3) | 0.073 |
| MVPA (min/d) | 5051 | 69.0 (35.1) | 2231 | 78.9 (38.8) | 2820 | 61.1 (29.6) | <0.001 |
| Weight (kg) | 5335 | 78.6 (16.5) | 2358 | 87.0 (14.6) | 2977 | 72.0 (14.9) | <0.001 |
| Height (cm) | 5337 | 170.9 (9.1) | 2358 | 178.5 (6.2) | 2979 | 164.8 (5.9) | <0.001 |
| Waist (cm) | 5306 | 91.7 (13.5) | 2344 | 97.4 (11.6) | 2962 | 87.2 (13.1) | <0.001 |
| Hip* (cm) | 5302 | 98.5 (93.5 - 104.5) | 2341 | 98.5 (94.5 - 103.5) | 2961 | 98.5 (93.0 - 106.0) | <0.001 |
| BMI (kg/m2) | 5335 | 26.8 (4.9) | 2358 | 27.3 (4.2) | 2977 | 26.5 (5.3) | <0.001 |
| WH ratio | 5302 | 0.92 (0.08) | 2341 | 0.98 (0.06) | 2961 | 0.87 (0.06) | <0.001 |
| HR (bpm) | 5301 | 70 (11) | 2343 | 68 (11) | 2958 | 72 (11) | <0.001 |
| SBP (mmHg) | 5304 | 125 (16) | 2344 | 131 (14) | 2960 | 121 (16) | <0.001 |
| DBP (mmHg) | 5304 | 85 (11) | 2344 | 87 (10) | 2960 | 83 (11) | <0.001 |
| Leucocytes* (E9/L) | 5342 | 5.4 (4.5 - 6.5) | 2362 | 5.3 (4.5 - 6.3) | 2980 | 5.4 (4.5 - 6.6) | 0.007 |
| Thrombocytes (E9/L) | 5333 | 252.5 (55.2) | 2359 | 235.1 (47.4) | 2974 | 266.3 (57.0) | <0.001 |
| Erythrocytes (E9/L) | 5342 | 4.7 (0.4) | 2362 | 4.9 (0.3) | 2980 | 4.5 (0.3) | <0.001 |
| Hemoglobin (g/L) | 5342 | 141.8 (11.4) | 2362 | 151.0 (7.6) | 2980 | 134.5 (8.3) | <0.001 |
| Fasting glucose* (mmol/l) | 5239 | 5.4 (5.1 - 5.8) | 2309 | 5.6 (5.3 - 6.0) | 2930 | 5.3 (5.0 - 5.6) | <0.001 |
| 2 h glucose in OGTT* (mmol/l) | 4515 | 5.6 (4.8 - 6.6) | 1971 | 5.7 (4.9 - 6.8) | 2544 | 5.5 (4.7 - 6.5) | <0.001 |
| HOMA-IR* | 5208 | 1.9 (1.2 - 2.9) | 2297 | 2.1 (1.4 - 3.3) | 2911 | 1.7 (1.2 - 2.6) | <0.001 |
| Fasting insulin* (mmol/l) | 5254 | 7.8 (5.3 - 11.6) | 2320 | 8.5 (5.7 - 12.6) | 2934 | 7.3 (5.1 - 10.6) | <0.001 |
| 2 h insulin in OGTT* (mmol/l) | 4521 | 42.7 (28.4 - 67.7) | 1975 | 43.0 (26.4 - 74.8) | 2546 | 42.6 (29.7 - 64.0) | 0.70 |
| Total cholesterol (mmol/L) | 5336 | 5.3 (0.9) | 2358 | 5.5 (1.0) | 2978 | 5.2 (0.8) | <0.001 |
| HDL cholesterol (mmol/L) | 5338 | 1.5 (0.4) | 2360 | 1.4 (0.3) | 2978 | 1.7 (0.4) | <0.001 |
| LDL cholesterol (mmol/L) | 5338 | 3.5 (0.9) | 2360 | 3.7 (0.9) | 2978 | 3.2 (0.8) | <0.001 |
| Triglycerides* (mmol/L) | 5338 | 1.0 (0.8 - 1.5) | 2360 | 1.3 (0.9 - 1.7) | 2978 | 0.9 (0.7 - 1.3) | <0.001 |
| Albumin (g/L) | 5338 | 45.0 (2.3) | 2360 | 45.9 (2.1) | 2978 | 44.3 (2.2) | <0.001 |
| Testosterone (nmol/L) | 5318 | 8.1 (9.3) | 2351 | 17.1 (6.8) | 2967 | 0.9 (0.3) | <0.001 |
| SHBG* (nmol/L) | 5321 | 41.4 (29.1 - 59.6) | 2353 | 32.0 (24.3 - 42.0) | 2968 | 52.7 (37.5 - 73.5) | <0.001 |

*Values as median (IQR) for non-normally distributed variables

n, number of; M, mean; SD, standard deviation; Mdn, median; IQR, interquartile range; MVPA, moderate-to-vigorous physical activity; min/d, minutes per day; BMI, body mass index; WH ratio, waist-to-hip ratio; SBP, systolic blood pressure; DBP, diastolic blood pressure, HR, heart rate; bpm, beats per minute; 2 h OGTT, 2-hour oral glucose tolerance test, HOMA-IR, homeostatic model assessment for insulin resistance; HDL, high-density lipoprotein; LDL, low-density lipoprotein; SHBG, sex-hormone binding globulin.

**Table S2. Heart rate variability and measures of baroreflex sensitivity of the study population.**

|  | All participants | | Males | | Females | |  |
| --- | --- | --- | --- | --- | --- | --- | --- |
| Variable (units) | n | M (SD) / Mdn (IQR) | n | M (SD) / Mdn (IQR) | n | M (SD) / Mdn (IQR) | *P* |
| Sitting |  |  |  |  |  |  |  |
| HR (bpm) | 5246 | 71.9 (11.2) | 2319 | 71.5 (11.9) | 2927 | 72.2 (10.7) | 0.017 |
| rMSSD (ms) | 5246 | 22.3 (14.6 - 33.0) | 2319 | 20.3 (13.3 - 30.3) | 2927 | 23.7 (15.7 - 35.1) | <0.001 |
| RRi (ms) | 5246 | 854.9 (133.4) | 2319 | 862.6 (142.7) | 2927 | 848.9 (125.2) | <0.001 |
| SDNN (ms) | 5246 | 35.0 (26.0 - 48.0) | 2319 | 35.0 (26.0 - 49.0) | 2927 | 36.0 (26.0 - 48.0) | 0.41 |
| ln(HF power) (ms^2^) | 5246 | 5.8 (1.0) | 2319 | 5.9 (1.0) | 2927 | 5.7 (1.0) | <0.001 |
| ln(LF power) (ms^2^) | 5246 | 5.3 (1.3) | 2319 | 5.1 (1.3) | 2927 | 5.4 (1.2) | <0.001 |
| LF/HF | 5244 | 1.6 (0.9 - 3.1) | 2318 | 2.3 (1.3 - 4.0) | 2926 | 1.2 (0.7 - 2.3) | <0.001 |
| SBP (mmHg) | 2437 | 119.1 (15.9) | 1130 | 122.6 (14.2) | 1307 | 116.1 (16.7) | <0.001 |
| LF_SBP_ (mmHg^2^) | 2437 | 5.5 (3.2 - 9.4) | 1130 | 5.7 (3.4 - 9.6) | 1307 | 5.4 (3.0 - 9.1) | 0.061 |
| BRS (ms/mmHg) | 2436 | 6.4 (4.6 - 8.9) | 1130 | 6.6 (4.8 - 9.4) | 1306 | 6.2 (4.4 - 8.5) | <0.001 |
| Standing |  |  |  |  |  |  |  |
| HR (bpm) | 5224 | 82.5 (12.8) | 2306 | 81.7 (13.2) | 2918 | 83.1 (12.4) | <0.001 |
| rMSSD (ms) | 5224 | 12.8 (8.5 - 19.1) | 2306 | 12.8 (8.5 - 18.8) | 2918 | 12.8 (8.5 - 19.4) | 0.44 |
| RRi (ms) | 5224 | 745.2 (117.0) | 2306 | 753.7 (122.0) | 2918 | 738.5 (112.5) | <0.001 |
| SDNN (ms) | 5224 | 32.7 (14.7) | 2306 | 31.0 (23.0 - 42.0) | 2918 | 29.0 (22.0 - 39.0) | <0.001 |
| ln(HF power) (ms^2^) | 5224 | 4.2 (1.3) | 2306 | 4.2 (1.3) | 2918 | 4.3 (1.3) | <0.001 |
| ln(LF power) (ms^2^) | 5224 | 5.4 (1.0) | 2306 | 5.6 (1.1) | 2918 | 5.3 (1.0) | <0.001 |
| LF/HF | 5217 | 3.3 (1.8 - 5.9) | 2303 | 4.4 (2.4 - 7.3) | 2914 | 2.7 (1.5 - 4.8) | <0.001 |
| SBP (mmHg) | 2420 | 117.4 (16.0) | 1120 | 121.1 (14.3) | 1300 | 114.3 (16.7) | <0.001 |
| LF_SBP_ (mmHg^2^) | 2420 | 151.1 (72.5 - 328.8) | 1120 | 189.8 (88.7 - 418.6) | 1300 | 123.5 (64.1 - 264.6) | <0.001 |
| BRS (ms/mmHg) | 2416 | 4.4 (3.1 - 6.2) | 1119 | 4.7 (3.2 - 6.7) | 1297 | 4.2 (3.0 - 5.8) | <0.001 |

n, number of; M, mean; SD, standard deviation; Mdn, median; IQR, interquartile range; ln, natural logarithm; HR, heart rate; rMSSD, root mean square of successive differences in RRi; RRi, R-R interval; SDNN, standard deviation of normal-to-normal interbeat intervals; HF, high-frequency; LF, low-frequency; LF/HF, low-to-high frequency ratio; SBP, systolic blood pressure; LF_SBP_, low-frequency SBP; BRS, baroreflex sensitivity.

**Table S3. Unadjusted associations of Hb levels within normal variation range with heart rate variability and measures of baroreflex sensitivity in all participants, males and females.**

|  | All participants | | | | | Males | | | | | Females | | | | |
| --- | --- | --- | --- | --- | --- | --- | --- | --- | --- | --- | --- | --- | --- | --- | --- |
| Variable | n | Beta | CIL | CIU | *P* | n | Beta | CIL | CIU | *P* | n | Beta | CIL | CIU | *P* |
| Sitting |  |  |  |  |  |  |  |  |  |  |  |  |  |  |  |
| HR (bpm) | 5246 | 0.11 | 0.08 | 0.14 | <0.001 | 2319 | 0.23 | 0.19 | 0.27 | <0.001 | 2927 | 0.16 | 0.13 | 0.20 | <0.001 |
| rMSSD (ms) | 5246 | -0.19 | -0.21 | -0.16 | <0.001 | 2319 | -0.19 | -0.23 | -0.15 | <0.001 | 2927 | -0.13 | -0.16 | -0.09 | <0.001 |
| RRi (ms) | 5246 | -0.10 | -0.12 | -0.07 | <0.001 | 2319 | -0.23 | -0.27 | -0.19 | <0.001 | 2927 | -0.16 | -0.20 | -0.13 | <0.001 |
| SDNN (ms) | 5246 | -0.09 | -0.11 | -0.06 | <0.001 | 2319 | -0.15 | -0.19 | -0.11 | <0.001 | 2927 | -0.09 | -0.12 | -0.05 | <0.001 |
| HF power (ms^2^) | 5246 | -0.20 | -0.23 | -0.17 | <0.001 | 2319 | -0.17 | -0.21 | -0.13 | <0.001 | 2927 | -0.12 | -0.15 | -0.08 | <0.001 |
| LF power (ms^2^) | 5246 | 0.01 | -0.02 | 0.03 | 0.63 | 2319 | -0.11 | -0.15 | -0.07 | <0.001 | 2927 | -0.06 | -0.09 | -0.02 | 0.002 |
| LF/HF | 5244 | 0.29 | 0.26 | 0.31 | <0.001 | 2318 | 0.12 | 0.08 | 0.16 | <0.001 | 2926 | 0.10 | 0.06 | 0.14 | <0.001 |
| SBP (mmHg) | 2437 | 0.19 | 0.16 | 0.23 | <0.001 | 1130 | 0.03 | -0.03 | 0.09 | 0.29 | 1307 | 0.10 | 0.05 | 0.15 | <0.001 |
| LF_SBP_ (mmHg^2^) | 2437 | 0.07 | 0.03 | 0.11 | <0.001 | 1130 | 0.05 | 0.00 | 0.11 | 0.065 | 1307 | 0.07 | 0.01 | 0.12 | 0.015 |
| BRS (ms/mmHg) | 2436 | -0.03 | -0.07 | 0.01 | 0.17 | 1130 | -0.11 | -0.17 | -0.06 | <0.001 | 1306 | -0.12 | -0.18 | -0.07 | <0.001 |
| Standing |  |  |  |  |  |  |  |  |  |  |  |  |  |  |  |
| HR (bpm) | 5224 | 0.09 | 0.06 | 0.12 | <0.001 | 2306 | 0.20 | 0.16 | 0.24 | <0.001 | 2918 | 0.17 | 0.14 | 0.21 | <0.001 |
| rMSSD (ms) | 5224 | -0.11 | -0.14 | -0.08 | <0.001 | 2306 | -0.14 | -0.18 | -0.10 | <0.001 | 2918 | -0.14 | -0.18 | -0.11 | <0.001 |
| RRi (ms) | 5224 | -0.09 | -0.11 | -0.06 | <0.001 | 2306 | -0.21 | -0.25 | -0.17 | <0.001 | 2918 | -0.17 | -0.21 | -0.14 | <0.001 |
| SDNN (ms) | 5224 | -0.04 | -0.07 | -0.01 | 0.005 | 2306 | -0.14 | -0.18 | -0.10 | <0.001 | 2918 | -0.11 | -0.14 | -0.07 | <0.001 |
| HF power (ms^2^) | 5224 | -0.13 | -0.15 | -0.10 | <0.001 | 2306 | -0.13 | -0.17 | -0.09 | <0.001 | 2918 | -0.13 | -0.16 | -0.09 | <0.001 |
| LF power (ms^2^) | 5224 | 0.03 | 0.01 | 0.06 | 0.014 | 2306 | -0.11 | -0.15 | -0.07 | <0.001 | 2918 | -0.10 | -0.13 | -0.06 | <0.001 |
| LF/HF | 5217 | 0.23 | 0.20 | 0.25 | <0.001 | 2303 | 0.05 | 0.01 | 0.09 | 0.017 | 2914 | 0.08 | 0.04 | 0.12 | <0.001 |
| SBP (mmHg) | 2420 | 0.20 | 0.16 | 0.24 | <0.001 | 1120 | 0.02 | -0.04 | 0.08 | 0.48 | 1300 | 0.11 | 0.05 | 0.16 | <0.001 |
| LF_SBP_ (mmHg^2^) | 2420 | 0.15 | 0.11 | 0.19 | <0.001 | 1120 | 0.08 | 0.02 | 0.14 | 0.010 | 1300 | 0.10 | 0.04 | 0.15 | <0.001 |
| BRS (ms/mmHg) | 2416 | -0.04 | -0.08 | 0.00 | 0.031 | 1119 | -0.12 | -0.17 | -0.06 | <0.001 | 1297 | -0.17 | -0.23 | -0.12 | <0.001 |

n, number of; M, mean; SD, standard deviation; Mdn, median; IQR, interquartile range; ln, natural logarithm; HR, heart rate; rMSSD, root mean square of successive differences in RRi; RRi, R-R interval; SDNN, standard deviation of normal-to-normal interbeat intervals; HF, high-frequency; LF, low-frequency; LF/HF, low-to-high frequency ratio; SBP, systolic blood pressure; LF_SBP_, low-frequency SBP; BRS, baroreflex sensitivity.

**Table S4.** **Age-, sex- and lifestyle-adjusted associations of Hb levels within normal variation range with heart rate variability and measures of baroreflex sensitivity in all participants, males and females.**

|  | All participants | | | | | Males | | | | | Females | | | | |
| --- | --- | --- | --- | --- | --- | --- | --- | --- | --- | --- | --- | --- | --- | --- | --- |
| Variable | n | Beta | CIL | CIU | *P* | n | Beta | CIL | CIU | *P* | n | Beta | CIL | CIU | *P* |
| Sitting |  |  |  |  |  |  |  |  |  |  |  |  |  |  |  |
| HR (bpm) | 4736 | 0.26 | 0.22 | 0.30 | <0.001 | 2067 | 0.22 | 0.17 | 0.26 | <0.001 | 2669 | 0.16 | 0.12 | 0.19 | <0.001 |
| rMSSD (ms) | 4736 | -0.21 | -0.25 | -0.17 | <0.001 | 2067 | -0.18 | -0.22 | -0.14 | <0.001 | 2669 | -0.12 | -0.16 | -0.09 | <0.001 |
| RRi (ms) | 4736 | -0.26 | -0.30 | -0.22 | <0.001 | 2067 | -0.22 | -0.26 | -0.18 | <0.001 | 2669 | -0.16 | -0.19 | -0.12 | <0.001 |
| SDNN (ms) | 4736 | -0.15 | -0.19 | -0.11 | <0.001 | 2067 | -0.14 | -0.19 | -0.10 | <0.001 | 2669 | -0.08 | -0.12 | -0.04 | <0.001 |
| HF power (ms^2^) | 4736 | -0.19 | -0.23 | -0.15 | <0.001 | 2067 | -0.16 | -0.21 | -0.12 | <0.001 | 2669 | -0.12 | -0.15 | -0.08 | <0.001 |
| LF power (ms^2^) | 4736 | -0.11 | -0.15 | -0.07 | <0.001 | 2067 | -0.11 | -0.15 | -0.07 | <0.001 | 2669 | -0.05 | -0.09 | -0.01 | 0.007 |
| LF/HF | 4736 | 0.15 | 0.11 | 0.19 | <0.001 | 2067 | 0.12 | 0.07 | 0.16 | <0.001 | 2669 | 0.11 | 0.07 | 0.15 | <0.001 |
| SBP (mmHg) | 2240 | 0.10 | 0.04 | 0.16 | 0.001 | 1027 | 0.02 | -0.04 | 0.09 | 0.43 | 1213 | 0.10 | 0.05 | 0.16 | <0.001 |
| LF_SBP_ (mmHg^2^) | 2240 | 0.10 | 0.04 | 0.16 | 0.001 | 1027 | 0.07 | 0.01 | 0.13 | 0.030 | 1213 | 0.07 | 0.01 | 0.13 | 0.014 |
| BRS (ms/mmHg) | 2239 | -0.17 | -0.23 | -0.11 | <0.001 | 1027 | -0.11 | -0.18 | -0.05 | <0.001 | 1212 | -0.12 | -0.18 | -0.07 | <0.001 |
| Standing |  |  |  |  |  |  |  |  |  |  |  |  |  |  |  |
| HR (bpm) | 4715 | 0.25 | 0.21 | 0.29 | <0.001 | 2057 | 0.19 | 0.15 | 0.23 | <0.001 | 2658 | 0.17 | 0.13 | 0.21 | <0.001 |
| rMSSD (ms) | 4715 | -0.19 | -0.23 | -0.15 | <0.001 | 2057 | -0.13 | -0.18 | -0.09 | <0.001 | 2658 | -0.14 | -0.18 | -0.10 | <0.001 |
| RRi (ms) | 4715 | -0.26 | -0.30 | -0.22 | <0.001 | 2057 | -0.20 | -0.24 | -0.16 | <0.001 | 2658 | -0.17 | -0.21 | -0.14 | <0.001 |
| SDNN (ms) | 4715 | -0.16 | -0.20 | -0.12 | <0.001 | 2057 | -0.13 | -0.17 | -0.08 | <0.001 | 2658 | -0.10 | -0.14 | -0.06 | <0.001 |
| HF power (ms^2^) | 4715 | -0.17 | -0.21 | -0.13 | <0.001 | 2057 | -0.11 | -0.16 | -0.07 | <0.001 | 2658 | -0.12 | -0.16 | -0.08 | <0.001 |
| LF power (ms^2^) | 4715 | -0.13 | -0.17 | -0.09 | <0.001 | 2057 | -0.10 | -0.14 | -0.05 | <0.001 | 2658 | -0.09 | -0.12 | -0.05 | <0.001 |
| LF/HF | 4710 | 0.10 | 0.06 | 0.14 | <0.001 | 2055 | 0.05 | 0.00 | 0.09 | 0.037 | 2655 | 0.09 | 0.05 | 0.13 | <0.001 |
| SBP (mmHg) | 2224 | 0.10 | 0.04 | 0.16 | 0.001 | 1018 | 0.01 | -0.05 | 0.07 | 0.80 | 1206 | 0.11 | 0.06 | 0.17 | <0.001 |
| LF_SBP_ (mmHg^2^) | 2224 | 0.15 | 0.09 | 0.21 | <0.001 | 1018 | 0.10 | 0.04 | 0.16 | 0.002 | 1206 | 0.11 | 0.05 | 0.16 | <0.001 |
| BRS (ms/mmHg) | 2220 | -0.20 | -0.26 | -0.14 | <0.001 | 1017 | -0.11 | -0.18 | -0.05 | <0.001 | 1203 | -0.16 | -0.22 | -0.11 | <0.001 |

The effect sizes were adjusted for age, sex, smoking status, alcohol consumption, and physical activity (Model 1). n, number of; M, mean; SD, standard deviation; Mdn, median; IQR, interquartile range; ln, natural logarithm; HR, heart rate; rMSSD, root mean square of successive differences in RRi; RRi, R-R interval; SDNN, standard deviation of normal-to-normal interbeat intervals; HF, high-frequency; LF, low-frequency; LF/HF, low-to-high frequency ratio; SBP, systolic blood pressure; LF_SBP_, low-frequency SBP; BRS, baroreflex sensitivity.

**Table S5. Age-, sex-, lifestyle- and metabolism-adjusted associations of Hb levels within normal variation range with heart rate variability and measures of baroreflex sensitivity in all participants, males and females.**

|  | All participants | | | | | Males | | | | | Females | | | | |
| --- | --- | --- | --- | --- | --- | --- | --- | --- | --- | --- | --- | --- | --- | --- | --- |
| Variable | n | Beta | CIL | CIU | *P* | n | Beta | CIL | CIU | *P* | n | Beta | CIL | CIU | *P* |
| Sitting |  |  |  |  |  |  |  |  |  |  |  |  |  |  |  |
| HR (bpm) | 4583 | 0.16 | 0.12 | 0.20 | <0.001 | 1992 | 0.14 | 0.10 | 0.18 | <0.001 | 2591 | 0.09 | 0.05 | 0.13 | <0.001 |
| rMSSD (ms) | 4583 | -0.11 | -0.15 | -0.07 | <0.001 | 1992 | -0.10 | -0.14 | -0.05 | <0.001 | 2591 | -0.06 | -0.10 | -0.02 | 0.003 |
| RRi (ms) | 4583 | -0.16 | -0.20 | -0.12 | <0.001 | 1992 | -0.14 | -0.18 | -0.10 | <0.001 | 2591 | -0.10 | -0.13 | -0.06 | <0.001 |
| SDNN (ms) | 4583 | -0.06 | -0.11 | -0.02 | 0.003 | 1992 | -0.07 | -0.12 | -0.03 | 0.001 | 2591 | -0.02 | -0.06 | 0.02 | 0.24 |
| HF power (ms^2^) | 4583 | -0.10 | -0.14 | -0.06 | <0.001 | 1992 | -0.09 | -0.13 | -0.04 | <0.001 | 2591 | -0.06 | -0.10 | -0.02 | 0.004 |
| LF power (ms^2^) | 4583 | -0.01 | -0.06 | 0.03 | 0.51 | 1992 | -0.03 | -0.07 | 0.02 | 0.219 | 2591 | 0.00 | -0.04 | 0.04 | 0.87 |
| LF/HF | 4583 | 0.12 | 0.08 | 0.16 | <0.001 | 1992 | 0.10 | 0.05 | 0.14 | <0.001 | 2591 | 0.08 | 0.04 | 0.12 | <0.001 |
| SBP (mmHg) | 2175 | 0.03 | -0.03 | 0.10 | 0.26 | 993 | -0.01 | -0.08 | 0.05 | 0.65 | 1182 | 0.05 | -0.01 | 0.11 | 0.081 |
| LF_SBP_ (mmHg^2^) | 2175 | 0.08 | 0.02 | 0.15 | 0.009 | 993 | 0.05 | -0.01 | 0.12 | 0.12 | 1182 | 0.06 | 0.00 | 0.13 | 0.036 |
| BRS (ms/mmHg) | 2167 | -0.03 | -0.09 | 0.03 | 0.28 | 989 | -0.01 | -0.07 | 0.05 | 0.64 | 1178 | -0.03 | -0.09 | 0.03 | 0.31 |
| Standing |  |  |  |  |  |  |  |  |  |  |  |  |  |  |  |
| HR (bpm) | 4565 | 0.19 | 0.15 | 0.23 | <0.001 | 1985 | 0.14 | 0.10 | 0.19 | <0.001 | 2580 | 0.13 | 0.09 | 0.17 | <0.001 |
| rMSSD (ms) | 4565 | -0.12 | -0.16 | -0.08 | <0.001 | 1985 | -0.08 | -0.13 | -0.04 | <0.001 | 2580 | -0.09 | -0.13 | -0.05 | <0.001 |
| RRi (ms) | 4565 | -0.20 | -0.24 | -0.15 | <0.001 | 1985 | -0.15 | -0.19 | -0.11 | <0.001 | 2580 | -0.13 | -0.17 | -0.09 | <0.001 |
| SDNN (ms) | 4565 | -0.08 | -0.12 | -0.04 | <0.001 | 1985 | -0.07 | -0.11 | -0.02 | 0.003 | 2580 | -0.05 | -0.09 | -0.01 | 0.021 |
| HF power (ms^2^) | 4565 | -0.10 | -0.14 | -0.05 | <0.001 | 1985 | -0.06 | -0.11 | -0.02 | 0.007 | 2580 | -0.07 | -0.11 | -0.03 | <0.001 |
| LF power (ms^2^) | 4565 | -0.04 | -0.08 | 0.00 | 0.072 | 1985 | -0.03 | -0.07 | 0.01 | 0.19 | 2580 | -0.03 | -0.07 | 0.01 | 0.18 |
| LF/HF | 4560 | 0.10 | 0.06 | 0.14 | <0.001 | 1983 | 0.06 | 0.01 | 0.10 | 0.018 | 2577 | 0.08 | 0.04 | 0.12 | <0.001 |
| SBP (mmHg) | 2159 | 0.02 | -0.04 | 0.08 | 0.50 | 984 | -0.04 | -0.11 | 0.03 | 0.23 | 1175 | 0.05 | -0.01 | 0.11 | 0.083 |
| LF_SBP_ (mmHg^2^) | 2159 | 0.12 | 0.05 | 0.18 | <0.001 | 984 | 0.08 | 0.02 | 0.15 | 0.014 | 1175 | 0.08 | 0.02 | 0.14 | 0.010 |
| BRS (ms/mmHg) | 2147 | -0.07 | -0.13 | -0.02 | 0.013 | 979 | -0.04 | -0.10 | 0.02 | 0.21 | 1168 | -0.06 | -0.12 | -0.01 | 0.030 |

The effect sizes were adjusted for sex, smoking status, alcohol consumption, physical activity, BMI, LDL cholesterol, SBP, and HOMA-IR. n, number of; M, mean; SD, standard deviation; Mdn, median; IQR, interquartile range; ln, natural logarithm; HR, heart rate; rMSSD, root mean square of successive differences in RRi; RRi, R-R interval; SDNN, standard deviation of normal-to-normal interbeat intervals; HF, high-frequency; LF, low-frequency; LF/HF, low-to-high frequency ratio; SBP, systolic blood pressure; LF_SBP_, low-frequency SBP; BRS, baroreflex sensitivity.

**Table S6. Age-, sex-, lifestyle-, metabolism-, and testosterone-adjusted associations of Hb levels within normal variation range with heart rate variability and baroreflex sensitivity measures in all participants, males and females.**

|  | All participants | | | | | Males | | | | | Females | | | | |
| --- | --- | --- | --- | --- | --- | --- | --- | --- | --- | --- | --- | --- | --- | --- | --- |
| Variable | n | Beta | CIL | CIU | *P* | n | Beta | CIL | CIU | *P* | n | Beta | CIL | CIU | *P* |
| Sitting |  |  |  |  |  |  |  |  |  |  |  |  |  |  |  |
| HR (bpm) | 4566 | 0.16 | 0.12 | 0.20 | <0.001 | 1985 | 0.14 | 0.10 | 0.18 | <0.001 | 2581 | 0.09 | 0.05 | 0.13 | <0.001 |
| rMSSD (ms) | 4566 | -0.11 | -0.15 | -0.07 | <0.001 | 1985 | -0.10 | -0.15 | -0.06 | <0.001 | 2581 | -0.06 | -0.10 | -0.02 | 0.003 |
| RRi (ms) | 4566 | -0.16 | -0.21 | -0.12 | <0.001 | 1985 | -0.14 | -0.18 | -0.1 | <0.001 | 2581 | -0.1 | -0.14 | -0.06 | <0.001 |
| SDNN (ms) | 4566 | -0.06 | -0.11 | -0.02 | 0.003 | 1985 | -0.07 | -0.12 | -0.03 | 0.001 | 2581 | -0.02 | -0.06 | 0.02 | 0.24 |
| HF power (ms^2^) | 4566 | -0.10 | -0.14 | -0.06 | <0.001 | 1985 | -0.09 | -0.13 | -0.05 | <0.001 | 2581 | -0.06 | -0.10 | -0.02 | 0.004 |
| LF power (ms^2^) | 4566 | -0.02 | -0.06 | 0.03 | 0.45 | 1985 | -0.03 | -0.07 | 0.01 | 0.17 | 2581 | 0.00 | -0.03 | 0.04 | 0.83 |
| LF/HF | 4566 | 0.12 | 0.08 | 0.16 | <0.001 | 1985 | 0.10 | 0.05 | 0.15 | <0.001 | 2581 | 0.08 | 0.04 | 0.13 | <0.001 |
| SBP (mmHg) | 2169 | 0.03 | -0.03 | 0.09 | 0.29 | 991 | -0.01 | -0.08 | 0.06 | 0.76 | 1178 | 0.05 | -0.01 | 0.11 | 0.097 |
| LF_SBP_ (mmHg^2^) | 2169 | 0.08 | 0.02 | 0.14 | 0.012 | 991 | 0.05 | -0.01 | 0.12 | 0.11 | 1178 | 0.06 | 0.00 | 0.12 | 0.043 |
| BRS (ms/mmHg) | 2161 | -0.03 | -0.09 | 0.03 | 0.27 | 987 | -0.02 | -0.08 | 0.04 | 0.56 | 1174 | -0.03 | -0.08 | 0.03 | 0.36 |
| Standing |  |  |  |  |  |  |  |  |  |  |  |  |  |  |  |
| HR (bpm) | 4548 | 0.19 | 0.15 | 0.23 | <0.001 | 1978 | 0.14 | 0.10 | 0.19 | <0.001 | 2570 | 0.13 | 0.09 | 0.17 | <0.001 |
| rMSSD (ms) | 4548 | -0.12 | -0.16 | -0.08 | <0.001 | 1978 | -0.08 | -0.13 | -0.04 | <0.001 | 2570 | -0.09 | -0.13 | -0.05 | <0.001 |
| RRi (ms) | 4548 | -0.20 | -0.24 | -0.15 | <0.001 | 1978 | -0.15 | -0.19 | -0.10 | <0.001 | 2570 | -0.13 | -0.17 | -0.09 | <0.001 |
| SDNN (ms) | 4548 | -0.08 | -0.12 | -0.04 | <0.001 | 1978 | -0.07 | -0.11 | -0.02 | 0.004 | 2570 | -0.05 | -0.09 | -0.01 | 0.017 |
| HF power (ms^2^) | 4548 | -0.10 | -0.14 | -0.06 | <0.001 | 1978 | -0.06 | -0.11 | -0.02 | 0.008 | 2570 | -0.07 | -0.11 | -0.03 | <0.001 |
| LF power (ms^2^) | 4548 | -0.04 | -0.08 | 0.00 | 0.066 | 1978 | -0.03 | -0.07 | 0.02 | 0.23 | 2570 | -0.03 | -0.07 | 0.01 | 0.16 |
| LF/HF | 4543 | 0.10 | 0.06 | 0.14 | <0.001 | 1976 | 0.06 | 0.01 | 0.11 | 0.015 | 2567 | 0.09 | 0.04 | 0.13 | <0.001 |
| SBP (mmHg) | 2153 | 0.02 | -0.04 | 0.08 | 0.54 | 982 | -0.04 | -0.10 | 0.03 | 0.28 | 1171 | 0.05 | -0.01 | 0.11 | 0.099 |
| LF_SBP_ (mmHg^2^) | 2153 | 0.11 | 0.05 | 0.17 | 0.001 | 982 | 0.09 | 0.02 | 0.15 | 0.010 | 1171 | 0.08 | 0.02 | 0.14 | 0.013 |
| BRS (ms/mmHg) | 2141 | -0.07 | -0.13 | -0.01 | 0.018 | 977 | -0.04 | -0.10 | 0.02 | 0.24 | 1164 | -0.06 | -0.12 | 0.00 | 0.037 |

The effect sizes were adjusted for sex, smoking status, alcohol consumption, physical activity, BMI, LDL cholesterol, SBP, HOMA-IR, and testosterone. n, number of; M, mean; SD, standard deviation; Mdn, median; IQR, interquartile range; ln, natural logarithm; HR, heart rate; rMSSD, root mean square of successive differences in RRi; RRi, R-R interval; SDNN, standard deviation of normal-to-normal interbeat intervals; HF, high-frequency; LF, low-frequency; LF/HF, low-to-high frequency ratio; SBP, systolic blood pressure; LF_SBP_, low-frequency SBP; BRS, baroreflex sensitivity.

**Table S7. Age-, sex-, lifestyle-, metabolism-, testosterone-, and fluid balance-adjusted associations of Hb levels within normal variation range with heart rate variability and baroreflex sensitivity measures in all participants, males and females.**

|  | All participants | | | | | Males | | | | | Females | | | | |
| --- | --- | --- | --- | --- | --- | --- | --- | --- | --- | --- | --- | --- | --- | --- | --- |
| Variable | n | Beta | CIL | CIU | *P* | n | Beta | CIL | CIU | *P* | n | Beta | CIL | CIU | *P* |
| Sitting |  |  |  |  |  |  |  |  |  |  |  |  |  |  |  |
| HR (bpm) | 4566 | 0.14 | 0.10 | 0.19 | <0.001 | 1985 | 0.13 | 0.09 | 0.17 | <0.001 | 2581 | 0.08 | 0.04 | 0.12 | <0.001 |
| rMSSD (ms) | 4566 | -0.10 | -0.14 | -0.05 | <0.001 | 1985 | -0.09 | -0.14 | -0.05 | <0.001 | 2581 | -0.05 | -0.09 | -0.01 | 0.017 |
| RRi (ms) | 4566 | -0.15 | -0.19 | -0.11 | <0.001 | 1985 | -0.13 | -0.18 | -0.09 | <0.001 | 2581 | -0.09 | -0.13 | -0.05 | <0.001 |
| SDNN (ms) | 4566 | -0.05 | -0.09 | -0.01 | 0.017 | 1985 | -0.07 | -0.11 | -0.02 | 0.004 | 2581 | -0.01 | -0.05 | 0.03 | 0.49 |
| HF power (ms^2^) | 4566 | -0.09 | -0.13 | -0.05 | <0.001 | 1985 | -0.08 | -0.13 | -0.04 | <0.001 | 2581 | -0.05 | -0.09 | -0.01 | 0.022 |
| LF power (ms^2^) | 4566 | -0.01 | -0.05 | 0.03 | 0.74 | 1985 | -0.03 | -0.07 | 0.02 | 0.251 | 2581 | 0.01 | -0.03 | 0.05 | 0.54 |
| LF/HF | 4566 | 0.11 | 0.07 | 0.16 | <0.001 | 1985 | 0.09 | 0.05 | 0.14 | <0.001 | 2581 | 0.08 | 0.04 | 0.12 | <0.001 |
| SBP (mmHg) | 2169 | 0.03 | -0.03 | 0.09 | 0.37 | 991 | -0.01 | -0.08 | 0.05 | 0.72 | 1178 | 0.05 | -0.01 | 0.10 | 0.13 |
| LF_SBP_ (mmHg^2^) | 2169 | 0.08 | 0.02 | 0.15 | 0.010 | 991 | 0.06 | -0.01 | 0.12 | 0.091 | 1178 | 0.06 | 0.00 | 0.12 | 0.042 |
| BRS (ms/mmHg) | 2161 | -0.03 | -0.09 | 0.03 | 0.29 | 987 | -0.02 | -0.08 | 0.05 | 0.60 | 1174 | -0.03 | -0.08 | 0.03 | 0.37 |
| Standing |  |  |  |  |  |  |  |  |  |  |  |  |  |  |  |
| HR (bpm) | 4548 | 0.17 | 0.13 | 0.21 | <0.001 | 1978 | 0.13 | 0.09 | 0.18 | <0.001 | 2570 | 0.11 | 0.07 | 0.15 | <0.001 |
| rMSSD (ms) | 4548 | -0.11 | -0.15 | -0.06 | <0.001 | 1978 | -0.07 | -0.12 | -0.03 | 0.002 | 2570 | -0.08 | -0.12 | -0.04 | <0.001 |
| RRi (ms) | 4548 | -0.18 | -0.22 | -0.14 | <0.001 | 1978 | -0.14 | -0.18 | -0.09 | <0.001 | 2570 | -0.12 | -0.16 | -0.08 | <0.001 |
| SDNN (ms) | 4548 | -0.07 | -0.11 | -0.02 | 0.002 | 1978 | -0.06 | -0.10 | -0.01 | 0.012 | 2570 | -0.04 | -0.08 | 0.00 | 0.056 |
| HF power (ms^2^) | 4548 | -0.09 | -0.13 | -0.05 | <0.001 | 1978 | -0.06 | -0.10 | -0.01 | 0.015 | 2570 | -0.07 | -0.11 | -0.03 | 0.001 |
| LF power (ms^2^) | 4548 | -0.02 | -0.07 | 0.02 | 0.25 | 1978 | -0.02 | -0.06 | 0.03 | 0.42 | 2570 | -0.02 | -0.06 | 0.02 | 0.40 |
| LF/HF | 4543 | 0.10 | 0.06 | 0.15 | <0.001 | 1976 | 0.06 | 0.01 | 0.11 | 0.012 | 2567 | 0.09 | 0.04 | 0.13 | <0.001 |
| SBP (mmHg) | 2153 | 0.02 | -0.05 | 0.08 | 0.61 | 982 | -0.04 | -0.10 | 0.03 | 0.27 | 1171 | 0.05 | -0.01 | 0.10 | 0.12 |
| LF_SBP_ (mmHg^2^) | 2153 | 0.12 | 0.06 | 0.19 | <0.001 | 982 | 0.10 | 0.03 | 0.17 | 0.003 | 1171 | 0.08 | 0.02 | 0.14 | 0.010 |
| BRS (ms/mmHg) | 2141 | -0.06 | -0.12 | 0.00 | 0.036 | 977 | -0.03 | -0.10 | 0.03 | 0.31 | 1164 | -0.05 | -0.11 | 0.00 | 0.060 |

The effect sizes were adjusted for sex, smoking status, alcohol consumption, physical activity, BMI, LDL cholesterol, SBP, HOMA-IR, testosterone levels, and albumin levels. n, number of; M, mean; SD, standard deviation; Mdn, median; IQR, interquartile range; ln, natural logarithm; HR, heart rate; rMSSD, root mean square of successive differences in RRi; RRi, R-R interval; SDNN, standard deviation of normal-to-normal interbeat intervals; HF, high-frequency; LF, low-frequency; LF/HF, low-to-high frequency ratio; SBP, systolic blood pressure; LF_SBP_, low-frequency SBP; BRS, baroreflex sensitivity.

**Table S8. Characteristics of the never-smokers and ever-smokers.**

|  | Never-smokers | | Ever-smokers | |  |
| --- | --- | --- | --- | --- | --- |
| Variable | n | M (SD) / Mdn (IQR) | n | M (SD) / Mdn (IQR) | *P* |
| Males, n (%) | 2713 | 1051 (38.7) | 2350 | 1156 (49.2) | <0.001 |
| Age (years) | 2713 | 46.6 (0.6) | 2350 | 46.6 (0.6) | 0.88 |
| Smoking status, n (%) | 2713 |  | 2350 |  | <0.001 |
| Never-smokers |  | 2713 (100.0) |  | 0 (0.0) |  |
| Ever-smoker |  | 0 (0.0) |  | 2350 (100.0) |  |
| Alcohol consumption, n (%) | 2711 |  | 2349 |  | <0.001 |
| 0-1 |  | 1014 (37.4) |  | 536 (22.8) |  |
| 2-5 |  | 1011 (37.3) |  | 769 (32.7) |  |
| 6 or more |  | 686 (25.3) |  | 1044 (44.4) |  |
| Use of antihypertensives, n (%) | 2713 | 332 (12.2) | 2350 | 363 (15.4) | <0.001 |
| MVPA (min/d) | 2601 | 70.6 (35.3) | 2216 | 67.3 (34.7) | <0.001 |
| Weight (kg) | 2710 | 76.7 (15.9) | 2347 | 80.5 (16.9) | <0.001 |
| Height (cm) | 2711 | 170.2 (9.3) | 2348 | 171.6 (8.9) | <0.001 |
| Waist (cm) | 2693 | 89.8 (13.0) | 2337 | 93.5 (13.7) | <0.001 |
| Hip* (cm) | 2692 | 98.0 (93.0 - 104.0) | 2336 | 99.0 (94.0 - 105.0) | <0.001 |
| WH ratio | 2692 | 0.89 (0.08) | 2336 | 0.93 (0.08) | <0.001 |
| BMI (kg/m2) | 2710 | 26.4 (4.8) | 2347 | 27.2 (4.9) | <0.001 |
| HR (bpm) | 2693 | 70 (11) | 2331 | 70 (11) | 0.063 |
| SBP (mmHg) | 2696 | 125 (16) | 2331 | 125 (16) | 0.80 |
| DBP (mmHg) | 2696 | 84 (10) | 2331 | 85 (11) | 0.049 |
| Leucocytes* (E9/L) | 2713 | 5.1 (4.4 - 6.2) | 2350 | 5.6 (4.8 - 6.8) | <0.001 |
| Thrombocytes (E9/L) | 2710 | 252.1 (56.0) | 2345 | 253.0 (54.8) | 0.56 |
| Erythrocytes (E9/L) | 2713 | 4.7 (0.4) | 2350 | 4.7 (0.4) | 0.009 |
| Hematocrit | 2713 | 0.42 (0.03) | 2350 | 0.42 (0.03) | <0.001 |
| Hemoglobin (g/L) | 2713 | 140.3 (11.4) | 2350 | 143.2 (11.3) | <0.001 |
| Fasting glucose* (mmol/l) | 2664 | 5.3 (5.1 - 5.7) | 2301 | 5.5 (5.2 - 5.9) | <0.001 |
| 2 h glucose in OGTT* (mmol/l) | 2375 | 5.6 (4.8 - 6.6) | 1940 | 5.6 (4.8 - 6.7) | 0.51 |
| HOMA-IR* | 2648 | 1.8 (1.2 - 2.7) | 2288 | 2.0 (1.3 - 3.1) | <0.001 |
| Fasting insulin* (mmol/l) | 2670 | 7.4 (5.1 - 11.0) | 2309 | 8.2 (5.4 - 12.0) | <0.001 |
| 2 h insulin in OGTT* (mmol/l) | 2378 | 42.4 (28.6 - 65.6) | 1943 | 43.1 (27.9 - 69.0) | 0.58 |
| Total cholesterol (mmol/L) | 2711 | 5.3 (0.9) | 2347 | 5.4 (0.9) | 0.016 |
| HDL cholesterol (mmol/L) | 2711 | 1.6 (0.4) | 2349 | 1.5 (0.4) | <0.001 |
| LDL cholesterol (mmol/L) | 2711 | 3.4 (0.9) | 2349 | 3.5 (1.0) | <0.001 |
| Triglycerides* (mmol/L) | 2711 | 1.0 (0.7 - 1.4) | 2349 | 1.1 (0.8 - 1.6) | <0.001 |
| Albumin (g/L) | 2711 | 44.9 (2.3) | 2349 | 45.1 (2.3) | <0.001 |
| Testosterone (nmol/L) | 2700 | 7.2 (9.0) | 2340 | 8.9 (9.5) | <0.001 |
| SHBG* (nmol/L) | 2700 | 43.3 (30.5 - 62.7) | 2343 | 39.4 (28.3 - 56.2) | <0.001 |

*Values as median (IQR) for non-normally distributed variables

n, number of; M, mean; SD, standard deviation; Mdn, median; IQR, interquartile range; MVPA, moderate-to-vigorous physical activity; min/d, minutes per day; BMI, body mass index; WH ratio, waist-to-hip ratio; SBP, systolic blood pressure; DBP, diastolic blood pressure, HR, heart rate; bpm, beats per minute; 2 h OGTT, 2-hour oral glucose tolerance test, HOMA-IR, homeostatic model assessment for insulin resistance; HDL, high-density lipoprotein; LDL, low-density lipoprotein; SHBG, sex-hormone binding globulin.

**Table S9. Heart rate variability and measures of baroreflex sensitivity in never-smokers and ever-smokers.**

|  | Never-smokers | | Ever-smokers | |  |
| --- | --- | --- | --- | --- | --- |
| Variable (units) | n | M (SD) / Mdn (IQR) | n | M (SD) / Mdn (IQR) | *P* |
| Sitting |  |  |  |  |  |
| HR (bpm) | 2664 | 71.4 (11.1) | 2312 | 72.3 (11.4) | 0.007 |
| rMSSD (ms) | 2664 | 23.1 (15.5 - 33.5) | 2312 | 21.5 (13.6 - 32.2) | <0.001 |
| RRi (ms) | 2664 | 860.4 (133.3) | 2312 | 850.6 (133.1) | 0.01 |
| SDNN (ms) | 2664 | 36.0 (27.0 - 49.0) | 2312 | 34.0 (25.0 - 47.0) | <0.001 |
| ln(HF power) (ms^2^) | 2664 | 5.8 (1.0) | 2312 | 5.7 (1.1) | <0.001 |
| ln(LF power) (ms^2^) | 2664 | 5.4 (1.2) | 2312 | 5.2 (1.3) | <0.001 |
| LF/HF | 2663 | 1.5 (0.8 - 3.0) | 2311 | 1.7 (0.9 - 3.1) | 0.003 |
| SBP (mmHg) | 1238 | 120.1 (16.2) | 1107 | 118.1 (15.4) | 0.003 |
| LF_SBP_ (mmHg^2^) | 1238 | 5.8 (3.3 - 10.0) | 1107 | 5.3 (3.0 - 8.9) | 0.002 |
| BRS (ms/mmHg) | 1237 | 6.4 (4.6 - 9.1) | 1107 | 6.3 (4.5 - 8.8) | 0.35 |
| Standing |  |  |  |  |  |
| HR (bpm) | 2655 | 82.2 (12.7) | 2300 | 82.7 (12.8) | 0.16 |
| rMSSD (ms) | 2655 | 13.3 (8.9 - 19.5) | 2300 | 12.6 (7.9 - 18.7) | <0.001 |
| RRi (ms) | 2655 | 747.9 (117.2) | 2300 | 743.4 (117.1) | 0.18 |
| SDNN (ms) | 2655 | 31.0 (24.0 - 41.0) | 2300 | 29.0 (22.0 - 39.0) | <0.001 |
| ln(HF power) (ms^2^) | 2655 | 4.3 (1.2) | 2300 | 4.2 (1.3) | <0.001 |
| ln(LF power) (ms^2^) | 2655 | 5.5 (1.0) | 2300 | 5.3 (1.1) | <0.001 |
| LF/HF | 2654 | 3.3 (1.8 - 5.8) | 2295 | 3.3 (1.8 - 5.9) | 0.65 |
| SBP (mmHg) | 1233 | 118.0 (16.3) | 1095 | 116.8 (15.6) | 0.066 |
| LF_SBP_ (mmHg^2^) | 1233 | 163.7 (79.8 - 352.6) | 1095 | 138.8 (64.1 - 308.5) | <0.001 |
| BRS (ms/mmHg) | 1230 | 4.4 (3.2 - 6.2) | 1094 | 4.4 (3.0 - 6.3) | 0.68 |

n, number of; M, mean; SD, standard deviation; Mdn, median; IQR, interquartile range; ln, natural logarithm; HR, heart rate; rMSSD, root mean square of successive differences in RRi; RRi, R-R interval; SDNN, standard deviation of normal-to-normal interbeat intervals; HF, high-frequency; LF, low-frequency; LF/HF, low-to-high frequency ratio; SBP, systolic blood pressure; LF_SBP_, low-frequency SBP; BRS, baroreflex sensitivity.

**Table S10. Association of Hb levels within normal variation range with heart rate variability and baroreflex sensitivity measures in never-smokers and ever-smokers.**

|  | Never-smokers | | | | | Ever-smokers | | | | |
| --- | --- | --- | --- | --- | --- | --- | --- | --- | --- | --- |
| Variable | n | B | CIL | CIU | *P* | n | B | CIL | CIU | *P* |
| Sitting |  |  |  |  |  |  |  |  |  |  |
| HR (bpm) | 2476 | 0.12 | 0.07 | 0.17 | <0.001 | 2107 | 0.20 | 0.14 | 0.26 | <0.001 |
| rMSSD (ms) | 2476 | -0.07 | -0.13 | -0.02 | 0.011 | 2107 | -0.15 | -0.21 | -0.08 | <0.001 |
| RRi (ms) | 2476 | -0.12 | -0.18 | -0.07 | <0.001 | 2107 | -0.21 | -0.27 | -0.15 | <0.001 |
| SDNN (ms) | 2476 | -0.03 | -0.08 | 0.03 | 0.34 | 2107 | -0.10 | -0.17 | -0.04 | 0.001 |
| HF power (ms^2^) | 2476 | -0.07 | -0.12 | -0.01 | 0.018 | 2107 | -0.13 | -0.19 | -0.07 | <0.001 |
| LF power (ms^2^) | 2476 | 0.02 | -0.03 | 0.08 | 0.47 | 2107 | -0.05 | -0.11 | 0.01 | 0.090 |
| LF/HF | 2476 | 0.11 | 0.06 | 0.17 | <0.001 | 2107 | 0.13 | 0.07 | 0.19 | <0.001 |
| SBP (mmHg) | 1159 | 0.06 | -0.02 | 0.14 | 0.16 | 1016 | 0.01 | -0.08 | 0.10 | 0.84 |
| LF_SBP_ (mmHg^2^) | 1159 | 0.11 | 0.02 | 0.19 | 0.014 | 1016 | 0.05 | -0.04 | 0.15 | 0.26 |
| BRS (ms/mmHg) | 1156 | -0.02 | -0.10 | 0.06 | 0.57 | 1011 | -0.04 | -0.13 | 0.04 | 0.34 |
| Standing |  |  |  |  |  |  |  |  |  |  |
| HR (bpm) | 2470 | 0.15 | 0.10 | 0.21 | <0.001 | 2095 | 0.23 | 0.17 | 0.29 | <0.001 |
| rMSSD (ms) | 2470 | -0.09 | -0.15 | -0.03 | 0.002 | 2095 | -0.16 | -0.22 | -0.09 | <0.001 |
| RRi (ms) | 2470 | -0.04 | -0.09 | 0.02 | 0.22 | 2095 | -0.24 | -0.30 | -0.18 | <0.001 |
| SDNN (ms) | 2470 | -0.16 | -0.21 | -0.10 | <0.001 | 2095 | -0.13 | -0.19 | -0.06 | <0.001 |
| HF power (ms^2^) | 2470 | -0.07 | -0.13 | -0.01 | 0.014 | 2095 | -0.12 | -0.19 | -0.06 | <0.001 |
| LF power (ms^2^) | 2470 | 0.02 | -0.04 | 0.07 | 0.58 | 2095 | -0.10 | -0.16 | -0.04 | 0.001 |
| LF/HF | 2469 | 0.12 | 0.06 | 0.17 | <0.001 | 2091 | 0.07 | 0.01 | 0.14 | 0.025 |
| SBP (mmHg) | 1155 | 0.05 | -0.03 | 0.13 | 0.21 | 1004 | -0.02 | -0.11 | 0.07 | 0.74 |
| LF_SBP_ (mmHg^2^) | 1155 | 0.14 | 0.05 | 0.22 | 0.002 | 1004 | 0.09 | -0.01 | 0.18 | 0.068 |
| BRS (ms/mmHg) | 1149 | -0.03 | -0.11 | 0.05 | 0.51 | 998 | -0.13 | -0.22 | -0.04 | 0.004 |

The effect sizes were adjusted for sex, smoking status, alcohol consumption, physical activity, BMI, LDL cholesterol, SBP, and HOMA-IR. n, number of; M, mean; SD, standard deviation; Mdn, median; IQR, interquartile range; ln, natural logarithm; HR, heart rate; rMSSD, root mean square of successive differences in RRi; RRi, R-R interval; SDNN, standard deviation of normal-to-normal interbeat intervals; HF, high-frequency; LF, low-frequency; LF/HF, low-to-high frequency ratio; SBP, systolic blood pressure; LF_SBP_, low-frequency SBP; BRS, baroreflex sensitivity.

**Table S11. Characteristics of the antihypertensive naïve- and antihypertensive user populations**

|  | Antihypertensive naive | | Antihypertensive users | |  |
| --- | --- | --- | --- | --- | --- |
| Variable (units) | n | M (SD) / Mdn (IQR) | n | M (SD) / Mdn (IQR) | *P* |
| Males, n (%) | 4643 | 2031 (43.7) | 699 | 331 (14.0) | 0.073 |
| Age (years) | 4643 | 46.6 (0.6) | 699 | 46.7 (0.6) | 0.51 |
| Smoking status, n (%) | 4368 |  | 695 |  | <0.001 |
| Never-smokers |  | 2381 (54.5) |  | 332 (47.8) |  |
| Ever-smokers |  | 1987 (45.5) |  | 363 (52.2) |  |
| Alcohol consumption, n (%) | 4394 |  | 698 |  | 0.002 |
| 0-1 |  | 1327 (30.2) |  | 230 (33.0) |  |
| 2-5 |  | 1587 (36.1) |  | 204 (29.2) |  |
| 6 or more |  | 1480 (33.7) |  | 264 (37.8) |  |
| Use of antihypertensives, n (%) | 4643 | 0 (0.0) | 699 | 699 (100.0) | <0.001 |
| MVPA (min/d) | 4390 | 70.1 (35.4) | 661 | 61.4 (31.7) | <0.001 |
| Weight (kg) | 4638 | 77.3 (15.5) | 697 | 87.1 (20.0) | <0.001 |
| Height (cm) | 4638 | 170.9 (9.1) | 699 | 170.8 (9.4) | 0.74 |
| Waist (cm) | 4611 | 90.5 (12.8) | 695 | 99.7 (15.1) | <0.001 |
| Hip* (cm) | 4607 | 99.2 (9.0) | 695 | 105.0 (12.3) | <0.001 |
| WH ratio | 4607 | 0.95 (0.09) | 695 | 0.91 (0.08) | <0.001 |
| BMI (kg/m2) | 4638 | 26.4 (4.5) | 697 | 29.8 (6.2) | <0.001 |
| HR (bpm) | 4607 | 70 (11) | 694 | 71 (12) | 0.16 |
| SBP (mmHg) | 4609 | 124 (16) | 695 | 131 (16) | <0.001 |
| DBP (mmHg) | 4609 | 84 (10) | 695 | 89 (10) | <0.001 |
| Leucocytes* (E9/L) | 4643 | 5.3 (4.5 - 6.4) | 699 | 5.8 (4.8 - 7.1) | <0.001 |
| Thrombocytes (E9/L) | 4636 | 251.8 (54.8) | 697 | 256.9 (57.7) | 0.024 |
| Erythrocytes (E9/L) | 4643 | 4.7 (0.4) | 699 | 4.7 (0.4) | <0.001 |
| Hematocrit | 4643 | 0.42 (0.09) | 699 | 0.42 (0.03) | <0.001 |
| Hemoglobin (g/L) | 4643 | 141.5 (11.5) | 699 | 143.7 (11.0) | <0.001 |
| Fasting glucose* (mmol/l) | 4552 | 5.4 (5.1 - 5.7) | 687 | 5.6 (5.3 - 6.1) | <0.001 |
| 2 h glucose in OGTT* (mmol/l) | 3971 | 5.6 (4.7 - 6.6) | 544 | 6.1 (5.1 - 7.4) | <0.001 |
| HOMA-IR* | 4529 | 1.8 (1.2 - 2.7) | 679 | 2.7 (1.8 - 4.4) | <0.001 |
| Fasting insulin* (mmol/l) | 4569 | 7.4 (5.1 - 10.9) | 685 | 10.9 (7.4 - 16.2) | <0.001 |
| 2 h insulin in OGTT* (mmol/l) | 3977 | 41.2 (27.8 - 62.6) | 544 | 61.3 (36.6 - 125.8) | <0.001 |
| Total cholesterol (mmol/L) | 4637 | 5.4 (0.9) | 699 | 5.2 (1.0) | 0.001 |
| HDL cholesterol (mmol/L) | 4639 | 1.6 (0.4) | 699 | 1.4 (0.3) | <0.001 |
| LDL cholesterol (mmol/L) | 4639 | 3.5 (0.9) | 699 | 3.4 (0.9) | 0.63 |
| Triglycerides* (mmol/L) | 4639 | 1.0 (0.8 - 1.4) | 699 | 1.3 (0.9 - 1.8) | <0.001 |
| Albumin (g/L) | 4639 | 45.0 (2.3) | 699 | 45.4 (2.4) | <0.001 |
| Testosterone (nmol/L) | 4625 | 8.1 (9.4) | 693 | 7.7 (8.6) | 0.32 |
| SHBG (nmol/L) | 4626 | 42.6 (30.0 - 61.0) | 695 | 34.9 (24.1 - 48.2) | <0.001 |

*Values as median (IQR) for non-normally distributed variables

n, number of; M, mean; SD, standard deviation; Mdn, median; IQR, interquartile range; MVPA, moderate-to-vigorous physical activity; min/d, minutes per day; BMI, body mass index; WH ratio, waist-to-hip ratio; SBP, systolic blood pressure; DBP, diastolic blood pressure, HR, heart rate; bpm, beats per minute; 2 h OGTT, 2-hour oral glucose tolerance test, HOMA-IR, homeostatic model assessment for insulin resistance; HDL, high-density lipoprotein; LDL, low-density lipoprotein; SHBG, sex-hormone binding globulin.

**Table S12. Heart rate variability and measures of baroreflex sensitivity in antihypertensive naïve- and antihypertensive user populations**

|  | Antihypertensive naive | | Antihypertensive users | |  |
| --- | --- | --- | --- | --- | --- |
| Variable | n | M (SD) / Mdn (IQR) | n | M (SD) / Mdn (IQR) | *P* |
| Sitting |  |  |  |  |  |
| HR (bpm) | 4557 | 71.7 (11.1) | 689 | 72.9 (12.2) | 0.008 |
| rMSSD (ms) | 4557 | 22.8 (15.1 - 33.6) | 689 | 18.8 (11.9 - 29.2) | <0.001 |
| RRi (ms) | 4557 | 856.3 (131.7) | 689 | 845.8 (143.7) | 0.053 |
| SDNN (ms) | 4557 | 36.0 (27.0 - 49.0) | 689 | 31.0 (22.0 - 43.0) | <0.001 |
| ln(HF power) (ms^2^) | 4557 | 5.8 (1.0) | 689 | 5.4 (1.1) | <0.001 |
| ln(LF power) (ms^2^) | 4557 | 5.3 (1.3) | 689 | 5.0 (1.4) | <0.001 |
| LF/HF | 4556 | 1.6 (0.9 - 3.1) | 688 | 1.7 (0.9 - 3.0) | 0.83 |
| SBP (mmHg) | 2105 | 118.9 (16.0) | 332 | 120.1 (14.8) | 0.22 |
| LF_SBP_ (mmHg^2^) | 2105 | 5.6 (3.2 - 9.5) | 332 | 5.1 (2.9 - 9.0) | 0.098 |
| BRS (ms/mmHg) | 2104 | 6.6 (4.7 - 9.0) | 332 | 5.5 (3.9 - 7.5) | <0.001 |
| Standing |  |  |  |  |  |
| HR (bpm) | 4542 | 82.5 (12.7) | 682 | 82.1 (13.4) | 0.38 |
| rMSSD (ms) | 4542 | 13.1 (8.7 - 19.4) | 682 | 11.4 (7.0 - 17.7) | <0.001 |
| RRi (ms) | 4542 | 744.3 (115.6) | 682 | 751.2 (126.2) | 0.15 |
| SDNN (ms) | 4542 | 31.0 (23.0 - 41.0) | 682 | 27.0 (19.0 - 36.0) | <0.001 |
| ln(HF power) (ms^2^) | 4542 | 4.3 (1.3) | 682 | 4.0 (1.3) | <0.001 |
| ln(LF power) (ms^2^) | 4542 | 5.5 (1.0) | 682 | 5.1 (1.1) | <0.001 |
| LF/HF | 4536 | 3.3 (1.8 - 5.9) | 681 | 3.0 (1.7 - 5.6) | 0.027 |
| SBP (mmHg) | 2094 | 117.3 (16.2) | 326 | 118.5 (14.8) | 0.21 |
| LF_SBP_ (mmHg^2^) | 2094 | 159.0 (77.0 - 340.2) | 326 | 98.2 (45.9 - 234.8) | <0.001 |
| BRS (ms/mmHg) | 2091 | 4.5 (3.2 - 6.3) | 325 | 3.9 (2.7 - 5.7) | <0.001 |

n, number of; M, mean; SD, standard deviation; Mdn, median; IQR, interquartile range; ln, natural logarithm; HR, heart rate; rMSSD, root mean square of successive differences in RRi; RRi, R-R interval; SDNN, standard deviation of normal-to-normal interbeat intervals; HF, high-frequency; LF, low-frequency; LF/HF, low-to-high frequency ratio; SBP, systolic blood pressure; LF_SBP_, low-frequency SBP; BRS, baroreflex sensitivity.

**Table S13. Association of Hb levels within normal variation range with heart rate variability in antihypertensive naïve- and antihypertensive population**

|  | Antihypertensive naive | | | | | Antihypertensive users | | | | |
| --- | --- | --- | --- | --- | --- | --- | --- | --- | --- | --- |
| Variable | n | B | CIL | CIU | *P* | n | B | CIL | CIU | *P* |
| Sitting |  |  |  |  |  |  |  |  |  |  |
| HR (bpm) | 3958 | 0.17 | 0.13 | 0.21 | <0.001 | 625 | 0.10 | 0.00 | 0.2 | 0.051 |
| rMSSD (ms) | 3958 | -0.12 | -0.16 | -0.07 | <0.001 | 625 | -0.05 | -0.15 | 0.05 | 0.34 |
| RRi (ms) | 3958 | -0.17 | -0.22 | -0.13 | <0.001 | 625 | -0.10 | -0.20 | 0.00 | 0.043 |
| SDNN (ms) | 3958 | -0.08 | -0.12 | -0.03 | 0.001 | 625 | -0.01 | -0.11 | 0.10 | 0.92 |
| HF power (ms^2^) | 3958 | -0.11 | -0.15 | -0.06 | <0.001 | 625 | -0.05 | -0.15 | 0.05 | 0.30 |
| LF power (ms^2^) | 3958 | -0.03 | -0.07 | 0.02 | 0.227 | 625 | 0.05 | -0.05 | 0.14 | 0.36 |
| LF/HF | 3958 | 0.12 | 0.07 | 0.16 | <0.001 | 625 | 0.13 | 0.03 | 0.24 | 0.011 |
| SBP (mmHg) | 1871 | 0.01 | -0.05 | 0.08 | 0.68 | 304 | 0.12 | -0.03 | 0.26 | 0.11 |
| LF_SBP_ (mmHg^2^) | 1871 | 0.07 | 0.00 | 0.14 | 0.044 | 304 | 0.12 | -0.03 | 0.27 | 0.12 |
| BRS (ms/mmHg) | 1863 | -0.04 | -0.10 | 0.03 | 0.25 | 304 | -0.01 | -0.15 | 0.13 | 0.87 |
| Standing |  |  |  |  |  |  |  |  |  |  |
| HR (bpm) | 3946 | 0.21 | 0.16 | 0.25 | <0.001 | 619 | 0.10 | 0.00 | 0.20 | 0.061 |
| rMSSD (ms) | 3946 | -0.14 | -0.19 | -0.10 | <0.001 | 619 | -0.02 | -0.12 | 0.09 | 0.72 |
| RRi (ms) | 3946 | -0.21 | -0.26 | -0.17 | <0.001 | 619 | -0.10 | -0.21 | 0.00 | 0.046 |
| SDNN (ms) | 3946 | -0.10 | -0.14 | -0.05 | <0.001 | 619 | 0.00 | -0.10 | 0.11 | 0.94 |
| HF power (ms^2^) | 3946 | -0.11 | -0.16 | -0.06 | <0.001 | 619 | -0.04 | -0.15 | 0.06 | 0.44 |
| LF power (ms^2^) | 3946 | -0.06 | -0.11 | -0.02 | 0.009 | 619 | 0.05 | -0.05 | 0.15 | 0.31 |
| LF/HF | 3942 | 0.10 | 0.05 | 0.14 | <0.001 | 618 | 0.11 | 0.01 | 0.22 | 0.038 |
| SBP (mmHg) | 1860 | -0.01 | -0.07 | 0.06 | 0.85 | 299 | 0.13 | -0.02 | 0.27 | 0.08 |
| LF_SBP_ (mmHg^2^) | 1860 | 0.10 | 0.03 | 0.17 | 0.004 | 299 | 0.15 | -0.01 | 0.30 | 0.065 |
| BRS (ms/mmHg) | 1849 | -0.08 | -0.15 | -0.02 | 0.013 | 298 | -0.04 | -0.19 | 0.11 | 0.61 |

The effect sizes were adjusted for sex, smoking status, alcohol consumption, physical activity, BMI, LDL cholesterol, SBP, and HOMA-IR. n, number of; M, mean; SD, standard deviation; Mdn, median; IQR, interquartile range; ln, natural logarithm; HR, heart rate; rMSSD, root mean square of successive differences in RRi; RRi, R-R interval; SDNN, standard deviation of normal-to-normal interbeat intervals; HF, high-frequency; LF, low-frequency; LF/HF, low-to-high frequency ratio; SBP, systolic blood pressure; LF_SBP_, low-frequency SBP; BRS, baroreflex sensitivity.

**Table S14. Association of Hb levels within normal variation range with heart rate variability in never-smoker antihypertensive naïve- and ever-smoker antihypertensive user populations**

|  | Antihypertensive naive,  never-smokers | | | | | Antihypertensive users,  ever-smokers | | | | |
| --- | --- | --- | --- | --- | --- | --- | --- | --- | --- | --- |
|  |  |  |  |  |  |  |  |  |  |  |
| Variable | n | B | CIL | CIU | *P* | n | B | CIL | CIU | *P* |
| Sitting |  |  |  |  |  |  |  |  |  |  |
| HR (bpm) | 2172 | 0.13 | 0.07 | 0.19 | <0.001 | 321 | 0.12 | -0.03 | 0.26 | 0.11 |
| rMSSD (ms) | 2172 | -0.08 | -0.14 | -0.02 | 0.010 | 321 | -0.05 | -0.20 | 0.10 | 0.52 |
| RRi (ms) | 2172 | -0.13 | -0.19 | -0.07 | <0.001 | 321 | -0.12 | -0.26 | 0.02 | 0.10 |
| SDNN (ms) | 2172 | -0.04 | -0.10 | 0.02 | 0.22 | 321 | -0.03 | -0.18 | 0.12 | 0.73 |
| HF power (ms^2^) | 2172 | -0.08 | -0.14 | -0.02 | 0.014 | 321 | -0.07 | -0.22 | 0.08 | 0.36 |
| LF power (ms^2^) | 2172 | 0.01 | -0.05 | 0.07 | 0.72 | 321 | 0.05 | -0.10 | 0.20 | 0.55 |
| LF/HF | 2172 | 0.11 | 0.05 | 0.17 | <0.001 | 321 | 0.15 | 0.01 | 0.29 | 0.036 |
| SBP (mmHg) | 1025 | 0.06 | -0.03 | 0.15 | 0.18 | 170 | 0.20 | 0.00 | 0.40 | 0.049 |
| LF_SBP_ (mmHg^2^) | 1025 | 0.11 | 0.01 | 0.20 | 0.023 | 170 | 0.18 | -0.03 | 0.38 | 0.095 |
| BRS (ms/mmHg) | 1022 | -0.03 | -0.11 | 0.06 | 0.54 | 170 | -0.02 | -0.21 | 0.17 | 0.84 |
| Standing |  |  |  |  |  |  |  |  |  |  |
| HR (bpm) | 2168 | 0.17 | 0.11 | 0.23 | <0.001 | 317 | 0.10 | -0.04 | 0.25 | 0.17 |
| rMSSD (ms) | 2168 | -0.10 | -0.17 | -0.04 | 0.001 | 317 | 0.00 | -0.15 | 0.15 | 0.99 |
| RRi (ms) | 2168 | -0.17 | -0.23 | -0.11 | <0.001 | 317 | -0.10 | -0.25 | 0.04 | 0.16 |
| SDNN (ms) | 2168 | -0.06 | -0.12 | 0.00 | 0.060 | 317 | -0.03 | -0.18 | 0.12 | 0.70 |
| HF power (ms^2^) | 2168 | -0.08 | -0.15 | -0.02 | 0.008 | 317 | -0.05 | -0.21 | 0.10 | 0.49 |
| LF power (ms^2^) | 2168 | 0.00 | -0.06 | 0.06 | 0.95 | 317 | 0.05 | -0.10 | 0.20 | 0.53 |
| LF/HF | 2167 | 0.11 | 0.05 | 0.17 | <0.001 | 316 | 0.10 | -0.05 | 0.25 | 0.19 |
| SBP (mmHg) | 1023 | 0.05 | -0.04 | 0.13 | 0.29 | 167 | 0.20 | -0.01 | 0.40 | 0.057 |
| LF_SBP_ (mmHg^2^) | 1023 | 0.13 | 0.04 | 0.22 | 0.004 | 167 | 0.19 | -0.02 | 0.40 | 0.070 |
| BRS (ms/mmHg) | 1018 | -0.03 | -0.12 | 0.05 | 0.48 | 167 | -0.06 | -0.27 | 0.15 | 0.562 |

The effect sizes were adjusted for sex, smoking status, alcohol consumption, physical activity, BMI, LDL cholesterol, SBP, and HOMA-IR. n, number of; M, mean; SD, standard deviation; Mdn, median; IQR, interquartile range; ln, natural logarithm; HR, heart rate; rMSSD, root mean square of successive differences in RRi; RRi, R-R interval; SDNN, standard deviation of normal-to-normal interbeat intervals; HF, high-frequency; LF, low-frequency; LF/HF, low-to-high frequency ratio; SBP, systolic blood pressure; LF_SBP_, low-frequency SBP; BRS, baroreflex sensitivity.

**Figure S1. Associations of Hb levels within normal variation range with measures of heart rate variability and baroreflex sensitivity adjusted for testosterone and albumin levels in all participants, males and females.** Forest plot representing the effect size estimates and their 95 % CIs for 1 SD change in the parameter of interest per 1 SD change in Hb in all participants (black), males (blue), and females (red). The effect sizes were adjusted for sex (in case of all participants), age, smoking status, alcohol consumption, physical activity, BMI, LDL cholesterol, SBP, HOMA-IR and in (A) for testosterone levels, and in (B) for testosterone and albumin levels. BMI, body mass index; LDL, low-density lipoprotein; HOMA-IR, homeostatic model assessment for insulin resistance; HR, heart rate; rMSSD, root mean square of successive differences in RRi; RRi, R-R interval; SDNN, standard deviation of normal-to-normal interbeat intervals, HF, high-frequency; LF, low-frequency; LF/HF, low-to-high frequency ratio; SBP, systolic blood pressure; LF_SBP_, low-frequency SBP; BRS, baroreflex sensitivity.
